# Manganese catalysed reduction of nitriles with amine boranes†

**DOI:** 10.1039/d4cy00813h

**Published:** 2024-07-24

**Authors:** Stefan Weber, Ines Blaha, Karl Kirchner

**Affiliations:** a Institute of Applied Synthetic Chemistry, TU Wien Getreidemarkt 9/163-AC A-1060 Wien Austria stefan.e163.weber@tuwien.ac.at karl.kirchner@tuwien.ac.at

## Abstract

The room temperature reduction of various nitriles using amine boranes (ABs) catalysed by a manganese(i) alkyl complex is described. Based on experimental findings, a plausible mechanistic scenario is presented. This includes the presence of two catalytic cycles, one for productive reduction of nitriles and one for hydrogen evolution.

## Abstract 2

The room temperature reduction of various nitriles using amine boranes catalysed by a manganese(i) alkyl complex is described. Based on experimental findings, a plausible mechanistic scenario is presented.

## Graphical abstract

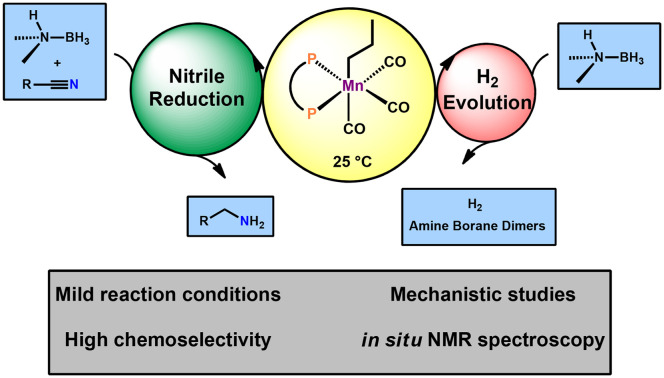

## Introduction

**Fig. 1:**
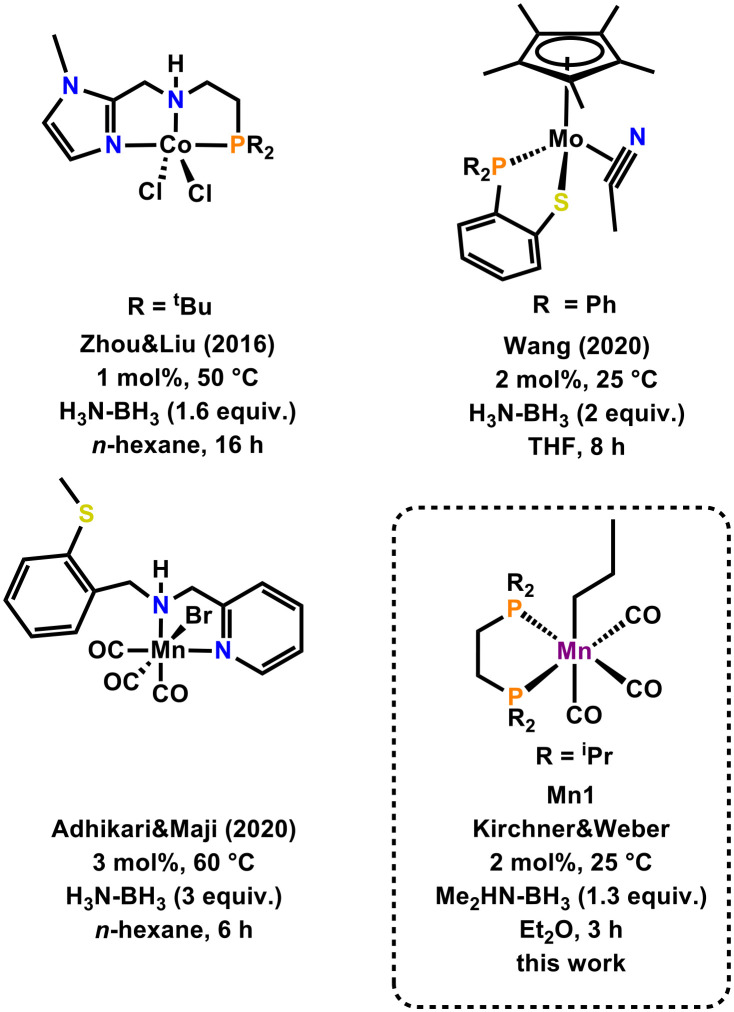
Well-defined base metal catalysts for the reduction of nitriles to primary amines using ABs.

The reduction of multiple bonds represents one of the most important transformations in organic chemistry. Apart from direct hydrogenation reactions employing hydrogen gas, transfer hydrogenation procedures can be utilised.^1^ Due to their high hydrogen content (up to 19.6 wt%), amine boranes (ABs) represent an interesting class of hydrogen donors for the reduction of unsaturated moieties.^2^ In order to avoid harsh reaction conditions or long reaction times, transition metal catalysts can be employed to facilitate increased reactivity.^3^ Noble metal complexes^4^ as well as base metal catalysts based on iron,^5^ cobalt,^6^ nickel,^7^ copper^8^ and very recently manganese^9^ may be utilized in such transformations. As far as the reduction of nitriles to primary amines is concerned, only little precedent is described using base-metal catalysts (Fig. 1). Zhou and Liu reported on the use of a PNN-based cobalt complex for the divergent reduction of nitriles to primary amines or secondary amines, depending on the choice of solvent.^10^ Later on, Wang employed a Mo(ii) complex supported by an anionic PS-ligand.^11^ Notably, this complex showed high reactivity at room temperature. Zhou utilized Cu(OTf)_2_ as a simple precatalyst in combination with an *in situ* generated amine borane in the presence of a super-stoichiometric amount of KO^*t*^Bu in aqueous media.^12^ Recently, Adhikari and Maji employed a Mn(i) complex, for the divergent reduction of nitriles to primary amines or secondary amines.^13^ A broad variety of nitriles could be successfully reduced under these conditions. However, three equivalents of AB had to be employed. Interestingly, all well-defined complexes shown in Fig. 1 contain a structural motif competent for metal–ligand cooperativity.^14^

Based on these examples, we questioned if such a motif is imperative for a productive reaction. Based on our previous findings on the reactivity of manganese alkyl carbonyl complexes for hydrogenation^15^ and hydrofunctionalization reactions^16^ we were curious if *fac*-[Mn(dippe)(CO)_3_(CH_2_CH_2_CH_3_)] (dippe = 1,2-bis(di-*iso*-propylphosphino)ethane) (**Mn1**) could be a competent catalyst for the afore described reaction. Herein we report on the application of **Mn1** as a pre-catalyst for the reduction of nitriles under mild conditions and short reaction times. Furthermore, we present mechanistic studies and propose a plausible scenario.

## Results and discussion

We began our investigations by choosing 4-fluorobenzonitrile as a model substrate in combination with various boranes as hydrogen source and **Mn1** as pre-catalyst. Selected optimization reactions are depicted in Table 1, for details see ESI.† High reactivity could be observed for various ABs at room temperature within 3 hours. This represents a rare example for the reduction of nitriles with ABs at room temperature in aprotic solvents.^11^ Dimethylamine borane (DMAB) performed best under these reaction conditions. We attribute this to high solubility of DMAB in organic solvents in comparison to methylamine borane and ammonia borane. Interestingly, no reactivity was observed for trimethylamine borane. This underscores the crucial role of the N–H motif in ABs for this reaction. Furthermore, no productivity was observed for pinacol borane (HBPin).

**Table 1:** Optimization reaction for the reduction of 4-fluorobenzonitrile by boranes catalyzed by **Mn1**^a^

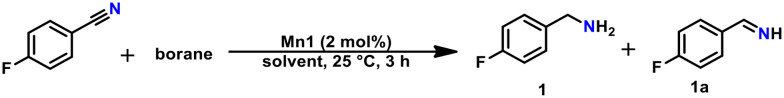				
Entry	Borane (equiv.)	Solvent	Conversion^b^ (%)	Ratio 1 : 1a^b^
1	HNMe_2_BH_3_ (2)	Et_2_O	>99	>99 : 1
2	H_2_NMeBH_3_ (2)	Et_2_O	43	98 : 2
3^c^	NMe_3_BH_3_ (2)	Et_2_O	—	—
4^c^	H_3_NBH_3_ (2)	Et_2_O	81	96 : 4
5^c^	HBPin (2)	Et_2_O	—	—
6	HNMe_2_BH_3_ (2)	THF	82	>99 : 1
7	HNMe_2_BH_3_ (2)	C_6_H_6_	>99	>99:1
8	HNMe_2_BH_3_ (2)	MeOH	—	—
9	**HNMe_2_BH_3_ (1.3)**	**Et_2_O**	**>99**	**>99 : 1**
10^d^	HNMe_2_BH_3_ (1)	Et_2_O	88	>99 : 1
11^e^	HNMe_2_BH_3_ (2)	Et_2_O	—	—

a Reaction conditions: 0.56 mmol 4-fluorobenzonitrile, **Mn1** (2 mol%) solvent (0.56 mL, 1 M), 25 °C, 3 h, Ar, closed vial.

b Determined by ^19^F{^1^H}-NMR spectroscopy.

c 24 h.

d 6 h.

e In absence of **Mn1**.

This is quite surprising, given our previous study wherein **Mn1** showed high productivity in the hydroboration of alkenes and alkynes with HBPin.^16*a*^ Silanes were also found to be unproductive for a reductive transformation. The implemented reduction procedure can be employed in a broad variety of solvents, whereas we found that alcohols lead to unproductive consumption of DMAB. Further optimization allowed us to decrease the equivalents of DMAB to 1.3 while still obtaining full conversion of nitrile. This is a significant lower amount than the vast majority of pervious reports in aprotic reaction media. Recently, Zhou and Liu reported on the Co catalysed reduction of nitriles, employing 1.6 equiv. of AB,^10^ whereas other reports utilize ≥2 equiv. of DMAB.^11–13^ In general, reducing the equivalents of reductant contributes to a more atom efficient reaction. However, lowering the amount of DMAB to one equivalent resulted in an incomplete reduction of the nitrile. Importantly, if the reaction is conducted in the absence of **Mn1** no conversion of nitrile is observed.

We then investigated the generality of the introduced reduction protocol for a variety of aromatic and aliphatic nitriles (Table 2). Halides, the ester functionality as well as furan or thiophene heterocycles are well tolerated. Lower productivity was observed in the presence of a nitro group. No or low conversion was detected for an aniline derivative as well as a pyridine substrate. High yields were achieved for dinitrile giving diamine 16. Remarkably, no reduction of the alkyne group (17) and only trace reduction (<5%) of the conjugated double bond in 18 was observed. This is quite remarkable, since **Mn1** was shown to efficiently reduce alkene^15*b*^ and alkynes^15*c*^ under hydrogenation conditions. This demonstrates that the choice of the hydrogen source can alter reactivity and selectivity of **Mn1**.

**Table 2:** Scope and limitation for nitrile reduction with DMAB catalysed by **Mn1**^a^

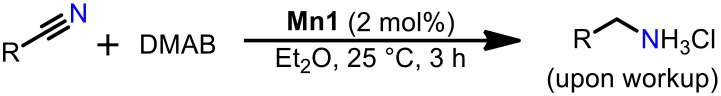
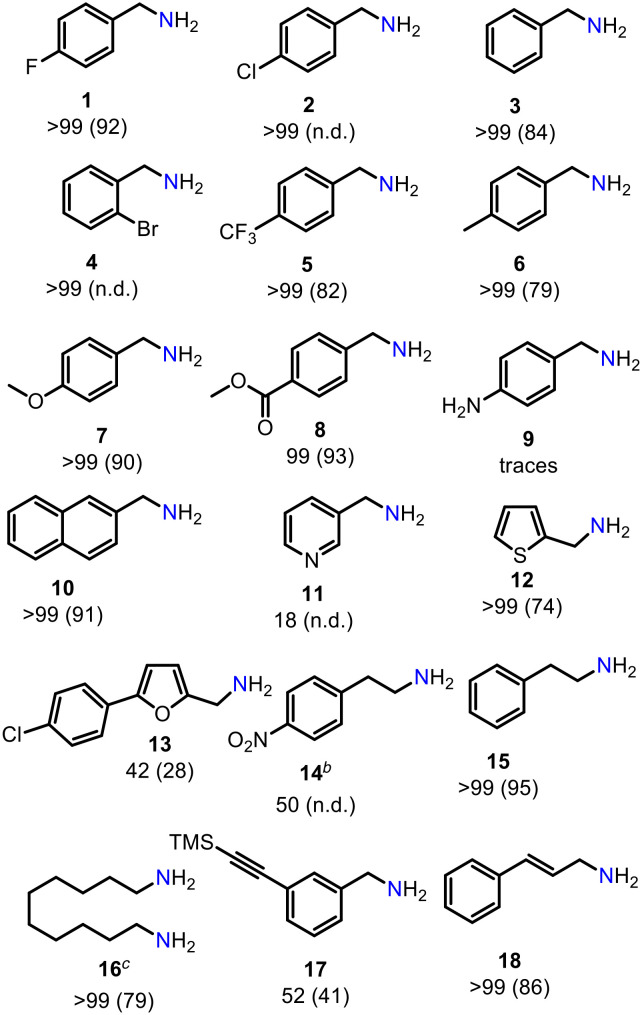

a Reaction conditions: 0.56 mmol nitrile (1 equiv.), 0.73 mmol DMAB (1.3 equiv.), **Mn1** (2 mol%), Et_2_O (1 M), 25 °C, 3 h Ar, closed vial. Conversion determined by GC-MS, isolated yields as ammonium chloride given in paratheses.

b THF, 50 °C.

c 1.4 mmol DMAB (2.5 equiv.), **Mn1** (2.5 mol%), 18 h. n.d. = not determined.

**Scheme 1:**
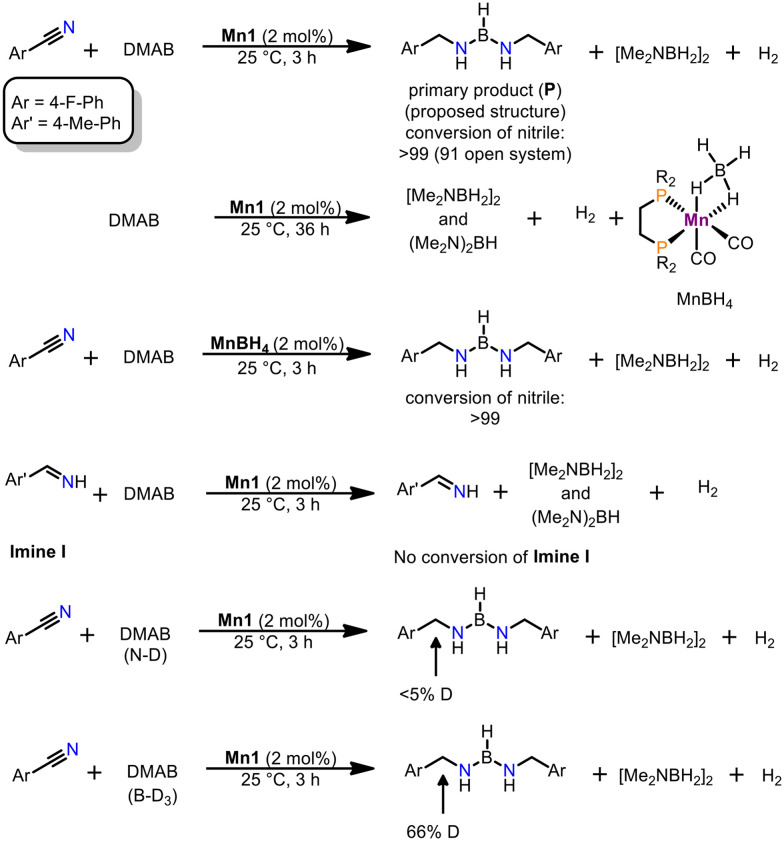
Mechanistic experiments for the reduction of nitriles by **Mn1**.

In order to gain insights in the reaction mechanism of the title reaction a series of experiments were conducted (Scheme 1). At first, analysis of the reaction mixture prior quenching was done. By employing ^11^B-NMR analysis, we were able to detect the cyclic dimer [Me_2_NBH_2_]_2_,^17^ a typical dehydrogenation product (triplet at 5.3 ppm) and a novel species, giving rise to a doublet centred at 29.0 ppm. Based on multinuclear NMR analysis and comparison with similar compounds presented in the literature,^18^ we assign the structure of the primary product (**P**) as depicted in Scheme 1. Unfortunately, we were unable to isolate **P** from the reaction mixture. This is attributed to complicated separation and its high sensitivity to moisture and air. It should be noted that the formation **P** (or related structures) is thus far unprecedented by the reduction of nitriles. Given the high sensitivity of **P**, it can be swiftly converted to free amines and eventually to ammonium salts, which were isolated in the substrate scope.

Next, we turned our attention to the interaction of **Mn1** with the reactants. While no reaction was observed between nitriles and **Mn1**, it reacts with an excess of DMAB to give the borohydride complex *cis*-[Mn(dippe)(CO)_3_(κ^2^-BH_4_)] (**MnBH**_**4**_)^19^ under release of hydrogen gas accompanied by the formation of typical amine–borane products upon dehydrogenation.^17,20^ Interestingly, this reaction is significantly slower (*ca.* 36 h for >95% conversion) than the reduction of nitriles (3 h for >99% conversion). If **MnBH**_**4**_ was directly used as pre-catalyst a similar reactivity was observed for the model reaction.

When the reaction was carried out in an open system, 91% conversion of nitrile was observed. This strongly favours a hydrogen transfer pathway over a dehydrogenation of DMAB followed by hydrogenation with released H_2_ gas. This is in line with an earlier report from our group, since direct hydrogenation requires harsher reaction conditions (100 °C, 50 bar H_2_) employing a similar manganese alkyl carbonyl complex.^15*a*^ Furthermore, formation of **P** is only consistent with a hydrogen- (and boron-) transfer mechanism and not with a classic hydrogenation reaction. Surprisingly, if **Imine I** was employed as substrate, no reaction was detected. This is consistent with reduction of nitriles to **P** within one catalytic cycle. Noteworthy, the presence of **Imine I** does not interfere with the dehydrogenation of DMAB. Given these observations, a stepwise reduction with substrate dissociation followed by recoordination seems unlikely. It should be noted that subjecting the cyclic dimer [Me_2_NBH_2_]_2_ as potential reductant, no conversion of neither nitrile nor **Imine I** could be observed. All of the above indicates that the dehydrogenation of DMAB is likely not coupled to productive nitrile reduction. We conclude that an interaction of nitrile, DMAB and catalyst is required for a productive process. Furthermore, deuterium labelling experiments were carried out. If DMAB-ND was employed as reductant, only traces of deuterium incorporation in the benzylic position was observed. The utilization of DMAB-BD_3_ gave a deuterium content of 66% in the benzylic position.

We then turned our attention to *in situ* NMR spectroscopy. Monitoring the reaction progress gave rise to a pronounced induction period followed by a pseudo-first order regime. This is consistent with a preequilibrium for the activation of **Mn1**. During the course of the reaction, two new sets of resonances for manganese-species were detected by ^31^P{^1^H} NMR spectroscopy. A singlet at 116.1 ppm corresponds to known tricarbonyl-hydride *fac*-[Mn(dippe)(CO)_3_(H)],^21^ which does not show any reactivity in the reduction of nitriles. The other manganese compound was tentatively assigned to *cis*-[Mn(dippe)(CO)_2_(nitrile)(H)] (**MnH**) which gives rise to two doublets centred at 129.3 ppm and 99.1 ppm in the ^31^P{^1^H}-NMR spectrum and a pseudo-triplet at −4.68 ppm in the ^1^H-NMR spectrum. This is in line with the related previously reported manganese hydride complex *cis*-[Mn(dippe)(CO)_2_(dmso)(H)].^22^ Deuterium labelling studies established that the B–H moiety in DMAB is the origin of the hydride rather than the proton of the N–H bond. Interestingly, the onset of catalysis was found to be dependent on the employed nitriles. More electron deficient nitriles initiated the catalytic reaction faster than electron-rich substrates. Furthermore, the reaction proceeds significantly faster with electron-poor nitriles.

These observations are in line with hydride transfer to the coordinated nitrile being the rate determining step during catalysis. This is also supported by a built up in the concentration of **MnH** during catalysis. Surprisingly, we could not detect **MnBH**_**4**_ during the course of the reaction, which seems to be an off-cycle species. However, we were able to demonstrate that **MnBH**_**4**_ reacts with nitriles to give **MnH** as an on-cycle intermediate. This interconversion may be fast under catalytic conditions, given the high concentration of substrate relative to **MnBH**_**4**_. Upon high conversion the fate of the active species is a variety of borohydride species.^16*a*^

**Scheme 2:**
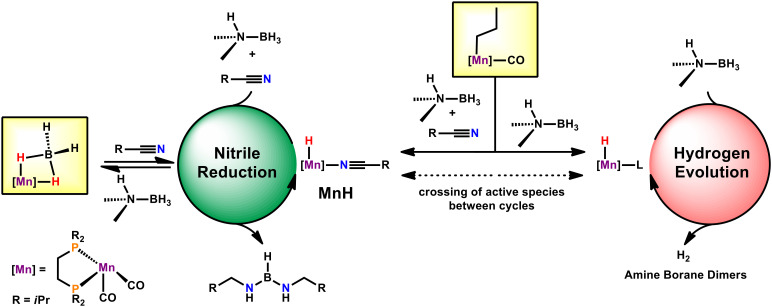
Proposed mechanistic scenario for the reduction of nitriles by DMAB catalysed by **Mn1**.

Based on these studies we suggest that two catalytic cycles are operative throughout catalysis (Scheme 2). One cycle presents the reduction of nitriles, whereas the other depicts the pathway for hydrogen evolution reaction (HER). Based on our experience with manganese alkyl carbonyl complexes, we propose activation of the catalyst by migratory insertion of the alkyl ligand into the adjacent CO.^15,16^ This can be initiated by DMAB alone, activating the catalyst for the hydrogen evolution pathway (Scheme 2, right cycle). Notably, the activation of **Mn1** solely by DMAB and the consecutive HER were shown to be rather sluggish. Alternatively, **Mn1** may also be activated by a combination of DMAB and nitrile substrate (Scheme 2, left side). This feeds into a productive cycle with superior turnover frequencies in comparison to the HER cycle. Within this pathway, **MnH** was found to be the resting state, presumably due to turnover-limiting hydride transfer to nitrile. Crossing over of the active species from the nitrile reduction cycle to the HER cycle (and *vice versa*) seems feasible. This is consistent with an earlier onset of HER and faster HER in presence of nitriles.

## Conclusions

In conclusion, we have introduced a manganese catalysed reduction of nitriles with amine boranes. This reaction takes place under mild reaction conditions and requires a reduced amount of ABs in comparison to previous reports. Based on mechanistic studies, we presented plausible reaction pathways. We propose two distinct catalytic cycles, one being productive for the reduction of nitriles by hydrogen- (and boron-) transfer, whereas the other represents a hydrogen evolution reaction. Future studies will be dedicated to the investigation of hydrogen release from amine boranes and (transfer) hydrogenation of unsaturated substrates by *in situ* generated hydrogen gas.

## Data availability

The authors of the manuscript entitled manganese catalysed reduction of nitriles with amine boranes by Stefan Weber, Ines Blaha and Karl Kirchner that all the data is available in ESI.† Original and processed data available in specific data formats can be provided on request from the corresponding authors. Raw NMR data associated with publication: Manganese Catalysed Reduction of Nitriles with Amine Boranes Stefan Weber, Ines Blaha and Kirchner, K. data can be directly processed Created with Bruker NMR software and will be deposited at the TU Wien repository (https://researchdata.tuwien.ac.at/).

## Author contributions

Stefan Weber contributed to conceptualization, investigation, methodology, visualization, and writing the original draft. Ines Blaha carried out experiments and the physical–chemical characterization. Karl Kirchner contributed to supervision, writing, review & editing, and funding acquisition.

## Conflicts of interest

There are no conflicts to declare.

## Supplementary Material

CY-014-D4CY00813H-s001
