# Supplementary material for: Manganese catalysed reduction of nitriles with amine boranes
Source: Catal Sci Technol. 2024 Jul 24;14(17):4843–7. doi: 10.1039/d4cy00813h (PMC11347915; doi:10.1039/d4cy00813h)
Supplement: CY-014-D4CY00813H-s001 [file CY-014-D4CY00813H-s001.pdf]

Supplementary Information (SI) for Catalysis Science & Technology.
This journal is © The Royal Society of Chemistry 2024

Manganese Catalysed Reduction of Nitriles with Amine Boranes

Stefan Weber,* Ines Blaha and Karl Kirchner*

Institute of Applied Synthetic Chemistry, TU Wien, Getreidemarkt 9/163-AC, A-1060 Wien, Austria

E-mail: karl.kirchner@tuwien.ac.at

E-Mail: stefan.e163.weber@tuwien.ac.at

Supporting Information

1 General Information 2

2 Experimental Procedures 2

3 Further optimization Reactions 3

4 Mechanistic Studies 4

5 Characterization of Organic Products 20

6 NMR Spectra of Products 21

7 References 34

1 General Information

All reactions were performed under an inert atmosphere of argon by using Schlenk techniques or in a MBraun inert-gas glovebox. The solvents were purified according to standard procedures. All -substrates and amine boranes were purchased from Sigma-Aldrich, Acros Organics or TCI and used as purchased without further purification. The deuterated solvents were purchased from Eurisotope and dried over 3 Å molecular sieves. Complexes **Mn1**,[1] *fac*-[Mn(dippe)$(CO)_3$H][2] and *cis*-[Mn(dippe)$(CO)_2$(κ^2-BH_4)] (**$MnBH_4$**)[3] were synthesized according to literature. ^{1}H and ^{13}C{^{1}H}, ^{11}B, ^{19}F and ^{31}P{^{1}H} NMR spectra were recorded on Bruker AVANCE-250, AVANCE-400, and AVANCE-600 spectrometers. ^{1}H and ^{13}C{^{1}H} NMR spectra were referenced internally to residual protio-solvent, and solvent resonances, respectively, and are reported relative to tetramethylsilane (δ = 0 ppm). ^{31}P{^{1}H} NMR spectra were referenced externally to H_3PO_4 (85%) (δ = 0 ppm). ^{11}B NMR spectra were referenced externally to $BF_3 \cdot Et_2O$ (15% in $CDCl_3$).

GC–MS analysis was conducted on an ISQ LT Single quadrupole MS (Thermo Fisher) directly interfaced to a TRACE 1300 Gas Chromatographic systems (Thermo Fisher), using a Rxi-5Sil MS (30 m, 0.25mm ID) cross-bonded dimethyl polysiloxane capillary column.

2 Experimental Procedures for the Reduction of Nitriles

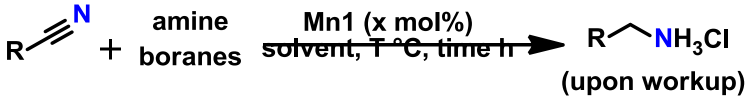

Inside an argon-flushed glovebox, a screwcap-vial (8 mL) was charged with **Mn1** (0.5-3 mol%), nitrile substrate (0.56 mmol, 1.0 equiv.), amine borane (0.56 mmol – 1.12 mmol, 1.0 -2.0 equiv.) and 0.56 mL solvent (1M) in this order. A stirring-bar was added, the vial was sealed, transferred outside the glovebox and the reaction mixture was heated to the indicated temperature (if required) and stirred for the indicated time. The reaction was quenched by exposure to air.

For optimization reaction, 50 µl of the sample were taken, diluted with 550 µl $CDCl_3$ and analysed by ^{19}F{^{1}H} NMR. In case of substrate scope, 10 µl of the sample was taken for GC-MS analysis.

Isolation of product

2.5 mL Et_2O and 1 mL of aqueous HCl (1M) were added. The biphasic system was stirred for approx. 30 minutes until gas evolution ceased. The pH of the aqueous phase was adjusted to approx. 10 using a NaOH solution (2M) and the phases were separated. The aqueous phase was thrice extracted with 2 mL Et_2O. The combined organic phases were washed once with water and once with brine. The organic phases were dried over Na_2SO_4 and the solvent was removed. The residue was redissolved in 5 mL Et_2O and ethereal HCl (1M) was added dropwise under stirring until precipitation was complete. The precipitate was collected by filtration and thoroughly washed with Et_2O. The ammonium salt was dried.

3 Further optimization Reactions

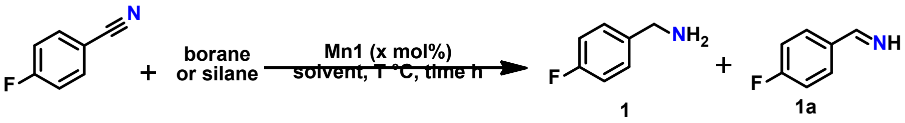

Entry	Catalyst loading [mol%]	Reductant (equiv.)	Solvent	Temperature [°C]	Reaction Time [h]	Conversion (%)	Ratio **1**:**1a**
1	3	**DMAB (2)**	THF	70	18	>99	>99:1
2	3	**Et_3SiH (2)**	THF	70	18	-	-
3	3	**$PhMe_2SiH$ (2)**	THF	70	18	-	-
4	3	**$PhSiH_3$**	THF	70	18	-	-
6	3	DMAB (2)	THF	**50**	18	>99	>99:1
7	**2**	DMAB (2)	THF	**50**	18	>99	>99:1
8	**2**	DMAB (2)	THF	**50**	18	>99	>99:1
9	**2**	DMAB (2)	C_6H_6	**50**	18	>99	>99:1
10	2	DMAB (2)	**THF**	**25**	18	>99	>99:1
11	2	DMAB (2)	**C_6H_6**	**25**	18	>99	>99:1
12	2	DMAB (2)	**Et_2O**	**25**	18	>99	>99:1
13	2	DMAB (2)	**DCM**	25	**3**	83	94:6
14	2	DMAB (2)	**Toluene**	25	**3**	91	97:3
15	2	DMAB (**1.6**)	Et_2O	25	3	>99	>99:1
16	2	DMAB (**1.4**)	Et_2O	25	3	>99	>99:1
17	2	DMAB (**1.2**)	Et_2O	25	3	94	98:2
18	**1**	DMAB (**1.3**)	Et_2O	25	3	89	97:3

Reaction conditions: 0.56 mmol 4-fluorobenzonitrile, **Mn1** (x mol%) solvent (0.56 mL, 1 M), conversion and ratio **1**:**1a** determined by $^{19}F\{^1H\}$-NMR spectroscopy.

4 Mechanistic Studies

4.1 Identification of the Primary Product (P)

Inside an argon-flushed glovebox, an NMR tube was charged with **Mn1** (0.0112 mmol, 2 mol%), 4-flurobenzonitrile (0.56 mmol, 1.0 equiv.), DMAB (0.73 mmol, 1.3 equiv.) and 0.56 mL C_6D_6 (1M). After 3 hours NMRs were recorded.

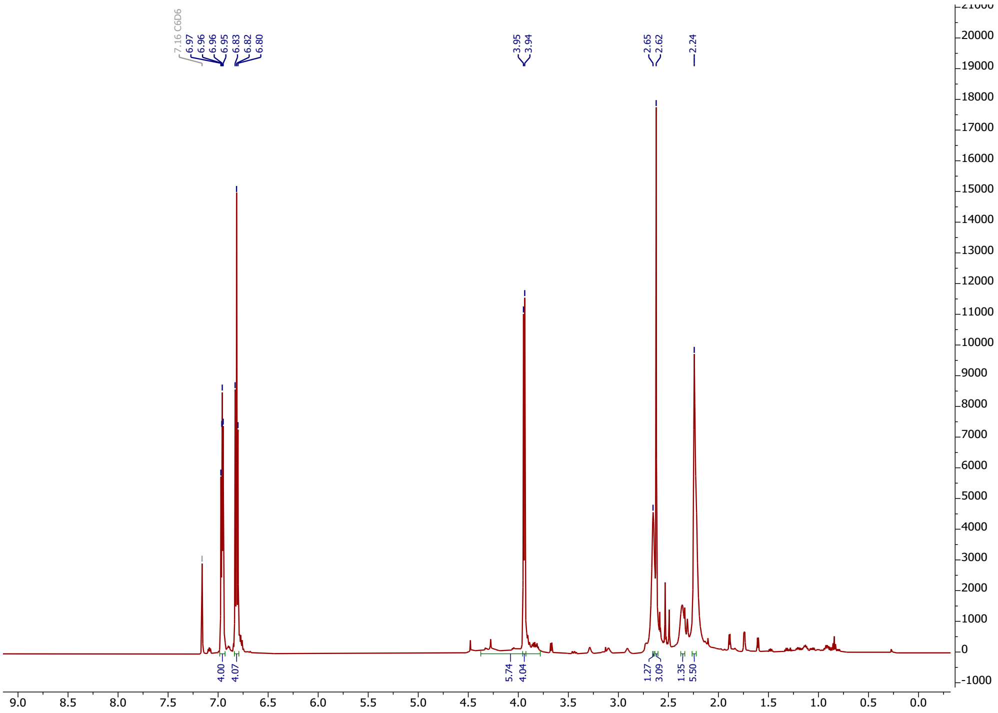

Figure S1: ^{1}H-NMR for the reduction 4-flurobenzonitrile with DMAB in presence of **Mn1**.

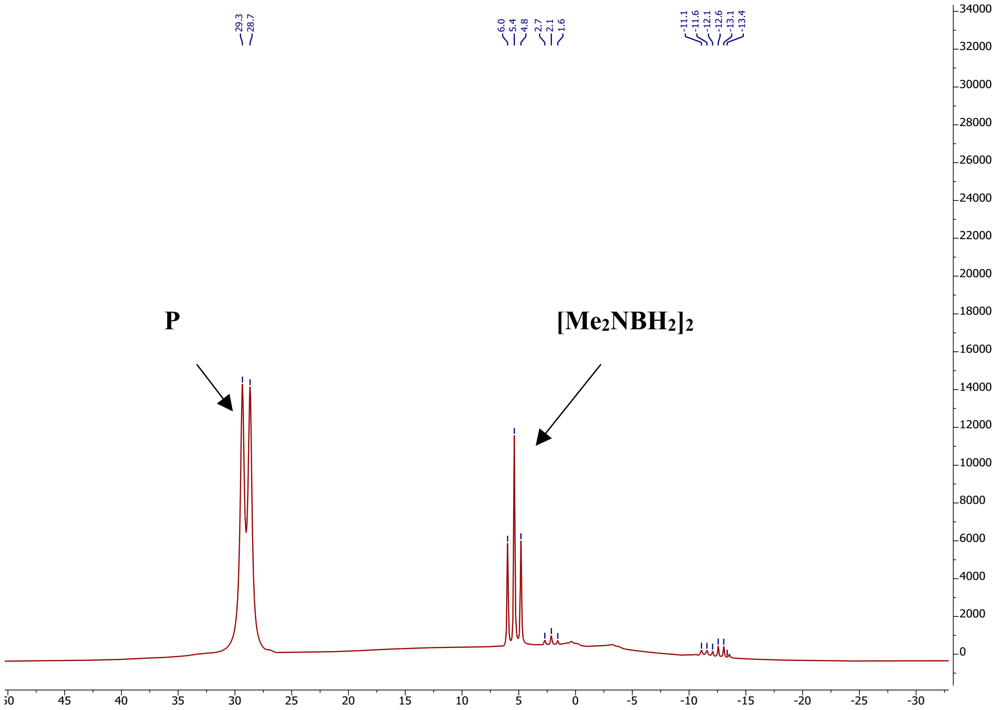

Figure S2: ^{11}B-NMR for the reduction 4-flurobenzonitrile with DMAB in presence of **Mn1**.

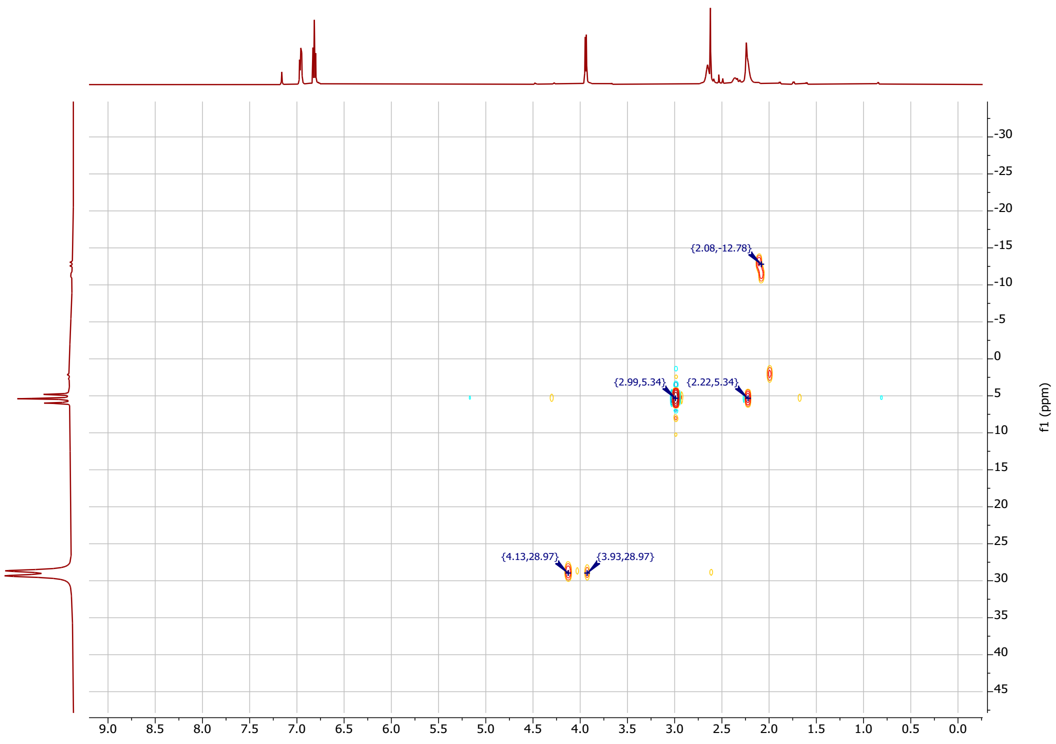

Figure S3: $^{1}H/^{11}B$-HSQC for the reduction 4-flurobenzonitrile with DMAB in presence of **Mn1**.

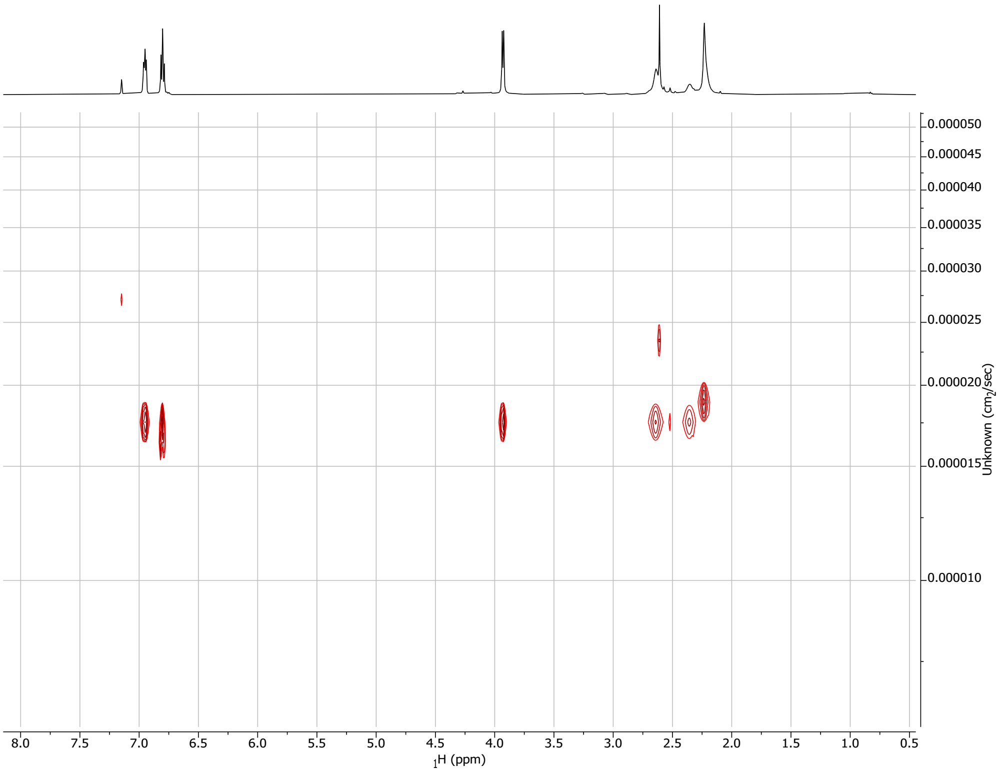

Figure S4: ^{1}H-DOSY for the reduction 4-flurobenzonitrile with DMAB in presence of **Mn1**.

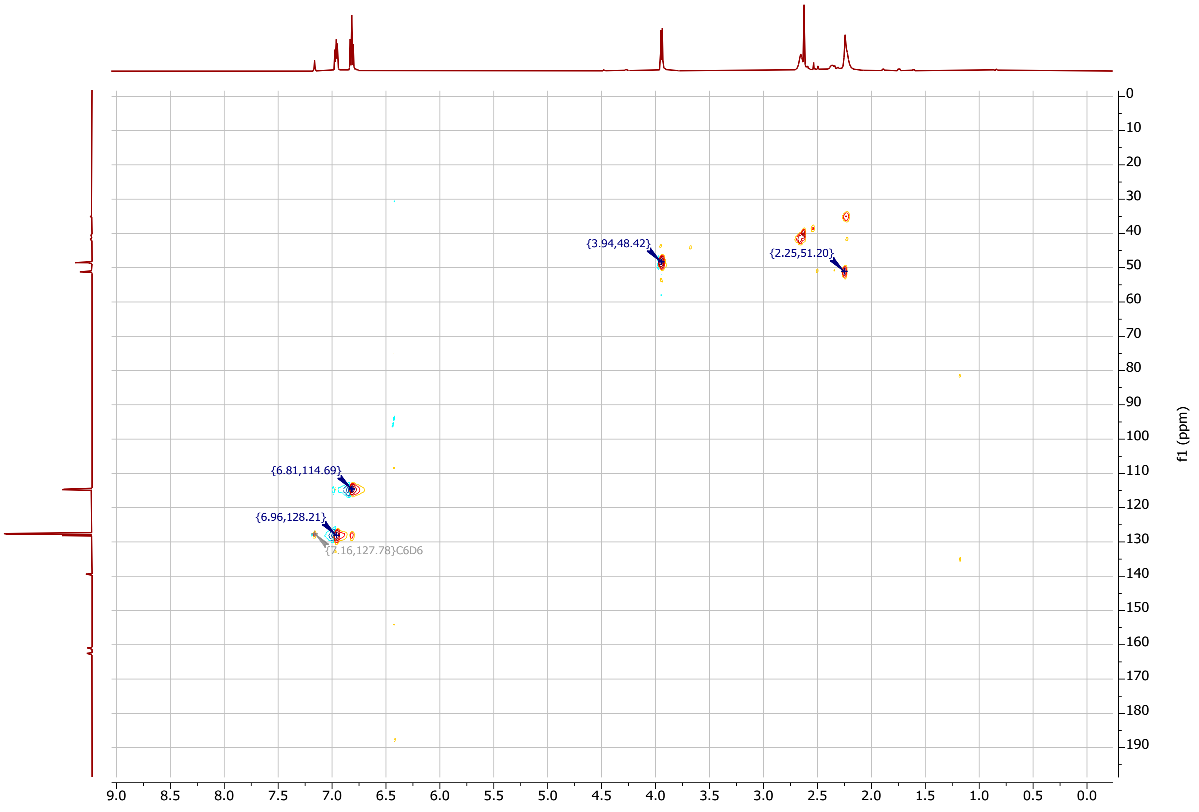

Figure S5: $^{1}H/^{13}C$-HSQC for the reduction 4-flurobenzonitrile with DMAB in presence of **Mn1**.

4.2 Mechanistic Experiments

4.2.1 Catalysis in absence of nitrile substrates

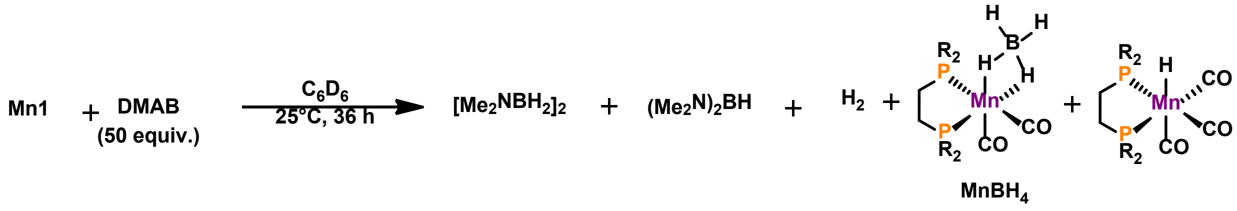

Inside an argon-flushed glovebox, an NMR tube was charged with **Mn1** (0.0112 mmol, 2 mol%) and DMAB (0.56 mmol, 1.0 equiv.) and 0.56 mL C_6D_6 (1M). NMRs were periodically recorded. Representative NMR data after 18 hours is shown below. 95% conversion of DMAB was detected after ~36 hours.

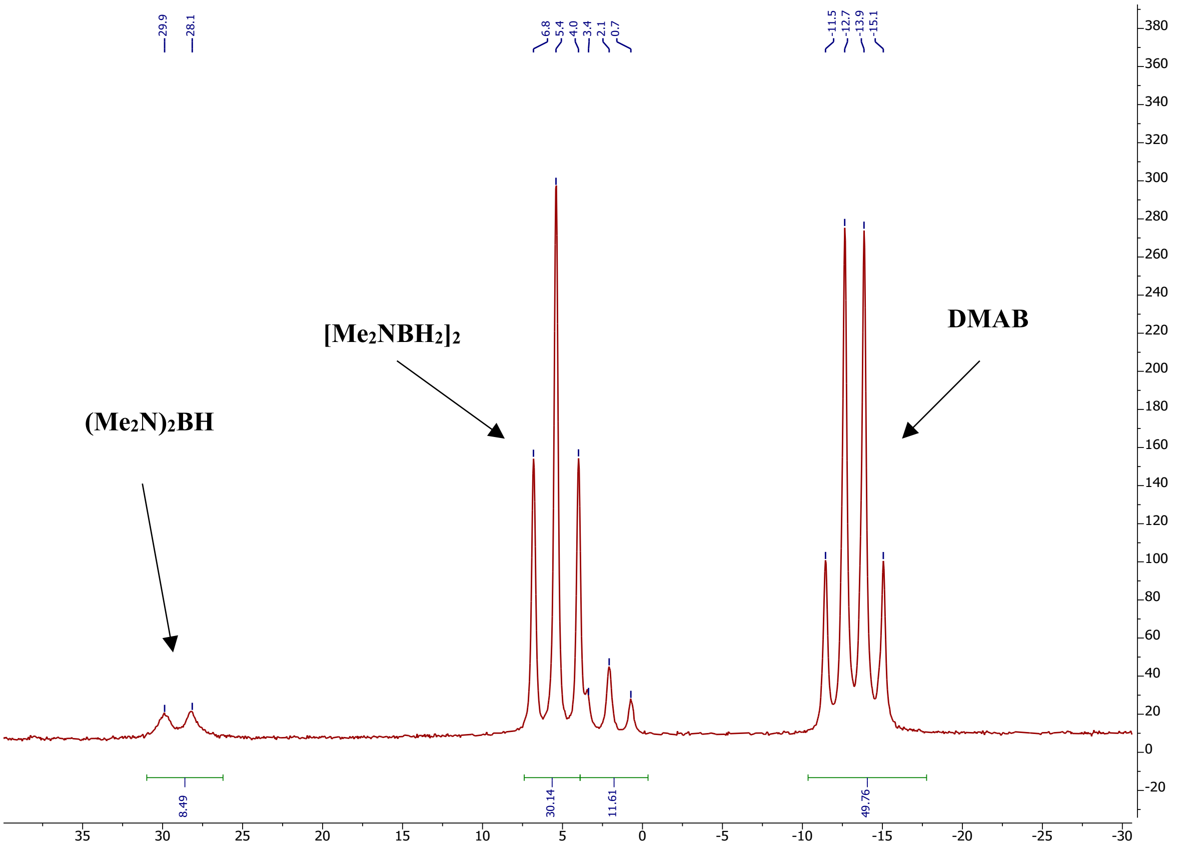

Figure S6: Representative ^{11}B-NMR for dehydrogenation of DMAB in presence of **Mn1** after 18 h.

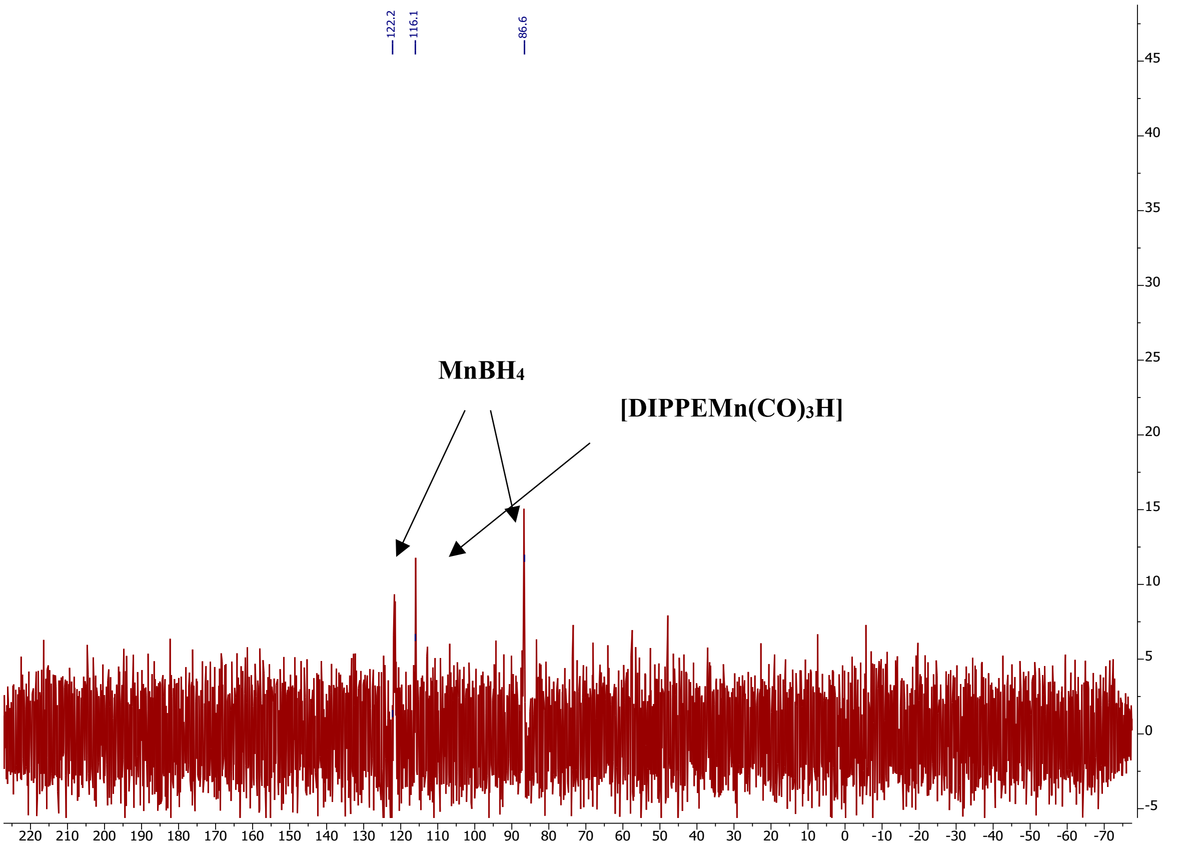

Figure S7: Representative $^{31}P\{^1H\}$-NMR for dehydrogenation of DMAB in presence of **Mn1** after 18 h.

4.2.2 Catalysis in an open system

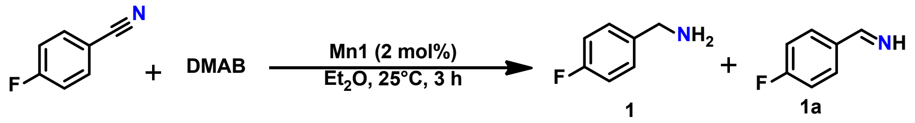

Inside an argon-flushed glovebox, a screw cap vial (8mL) was charged with **Mn1** (0.0112 mmol, 2 mol%), 4-flurobenzonitrile (0.56 mmol, 1.0 equiv.), DMAB (0.73 mmol, 1.3 equiv.) and 0.56 mL Et_2O (1M). The vial was not sealed and placed in a 30 mL Schlenk tube. The Schlenk tube was brought out of the glove box and opened to the Schlenkline. After 3 hours 50 µl of the sample were taken, diluted with 550 µl $CDCl_3$ and analysed by $^{19}F\{^1H\}$ NMR. 91 % conversion of nitrile substrate was detected (>99% was detected in a closed system).

4.2.3 Catalysis with Imine substrate

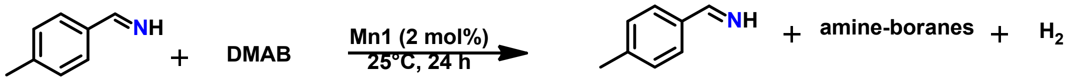

Inside an argon-flushed glovebox, an NMR tube was charged with **Mn1** (0.0112 mmol, 2 mol%), **Imine I** (generated from the iminium hydro chloride and 1.05 equiv. triethylamine before the reaction and used immediately) (0.56 mmol, 1.0 equiv.), DMAB (0.73 mmol, 1.3 equiv.) and 0.56 mL C_6D_6 (1M). NMR analysis was periodically conducted. No consumption of **Imine I** was observed over the course of 24 h. The dehydrogenation of DMAB is not affected by the presence of **Imine I.**

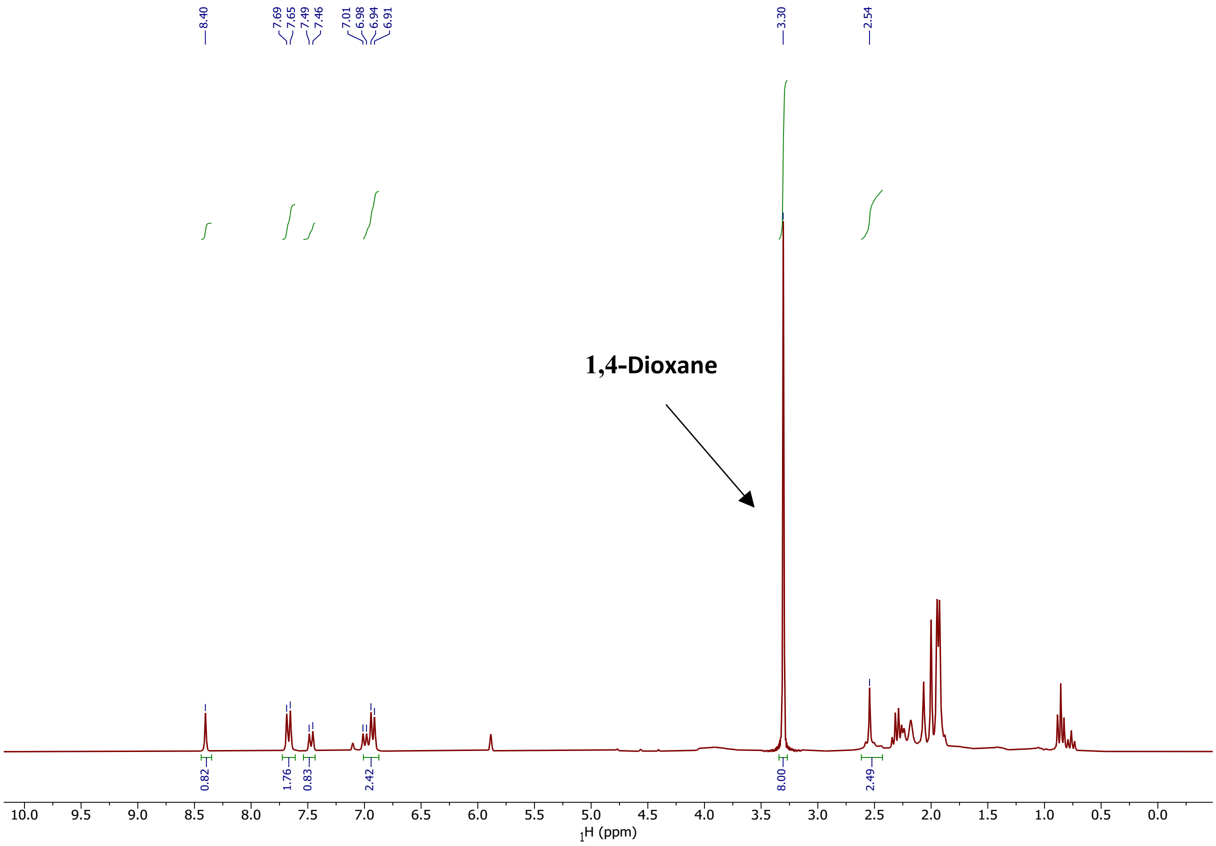

Figure S8: ^1H-NMR for attempted reduction of **Imine I** with DMAB in presence of **Mn1** after 24 h.

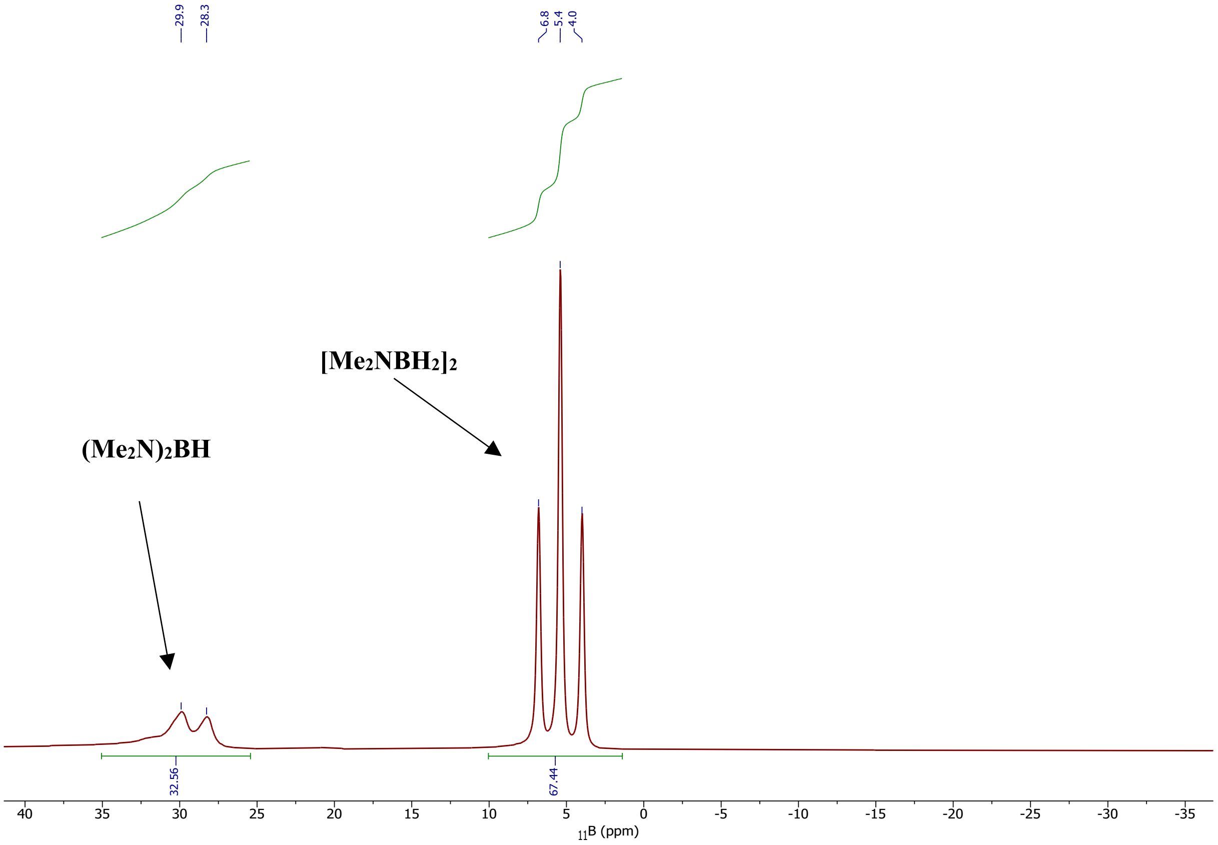

Figure S9: ^{11}B-NMR for attempted reduction of **Imine I** with DMAB in presence of **Mn1** after 24 h.

4.2.4 Deuterium labelling studies

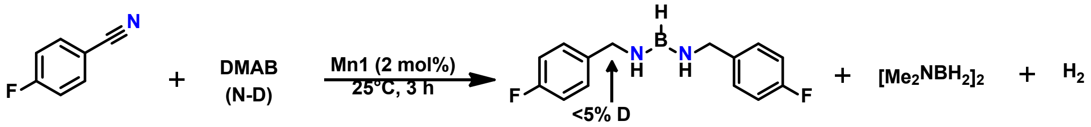

Inside an argon-flushed glovebox, an NMR tube was charged with **Mn1** (0.0112 mmol, 2 mol%), 4-flurobennzonitrile (0.56 mmol, 1.0 equiv.), DMAB *N-D* (~95 % D) (0.73 mmol, 1.3 equiv.) and 0.56 mL C_6D_6 (1M). NMR analysis was periodically conducted. 91% conversion was detected after 3 hours (>99% for DMAB N-H). Deuterium content was determined upon full conversion. <5% of deuterium incorporation was found in the benzylic position.

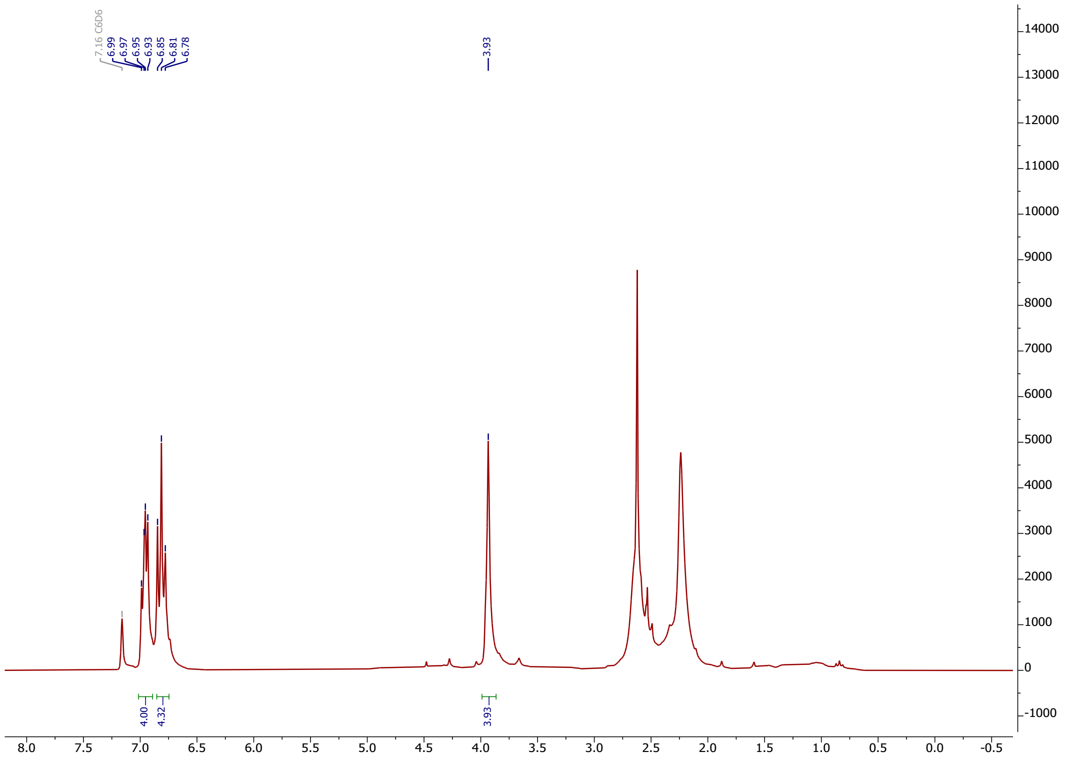

Figure S11: ^{1}H-NMR for reduction of 4-flurobenzonitrile with DMAB *N-D* in presence of **Mn1** after 5 h.

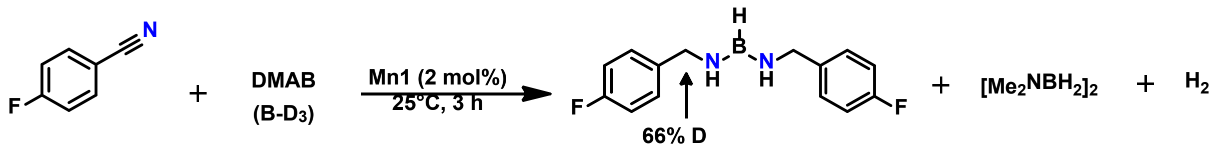

Inside an argon-flushed glovebox, an NMR tube was charged with **Mn1** (0.0112 mmol, 2 mol%), 4-flurobenzonitrile (0.56 mmol, 1.0 equiv.), DMAB *B-D_3* (~93 % D) (0.73 mmol, 1.3 equiv.) and 0.56 mL THF-*d8* (1M). NMR analysis was periodically conducted. 69 % conversion was detected after 3 hours (82% for DMAB B-H_3). Deuterium content was determined upon full conversion. 66% of deuterium incorporation was found in the benzylic position.

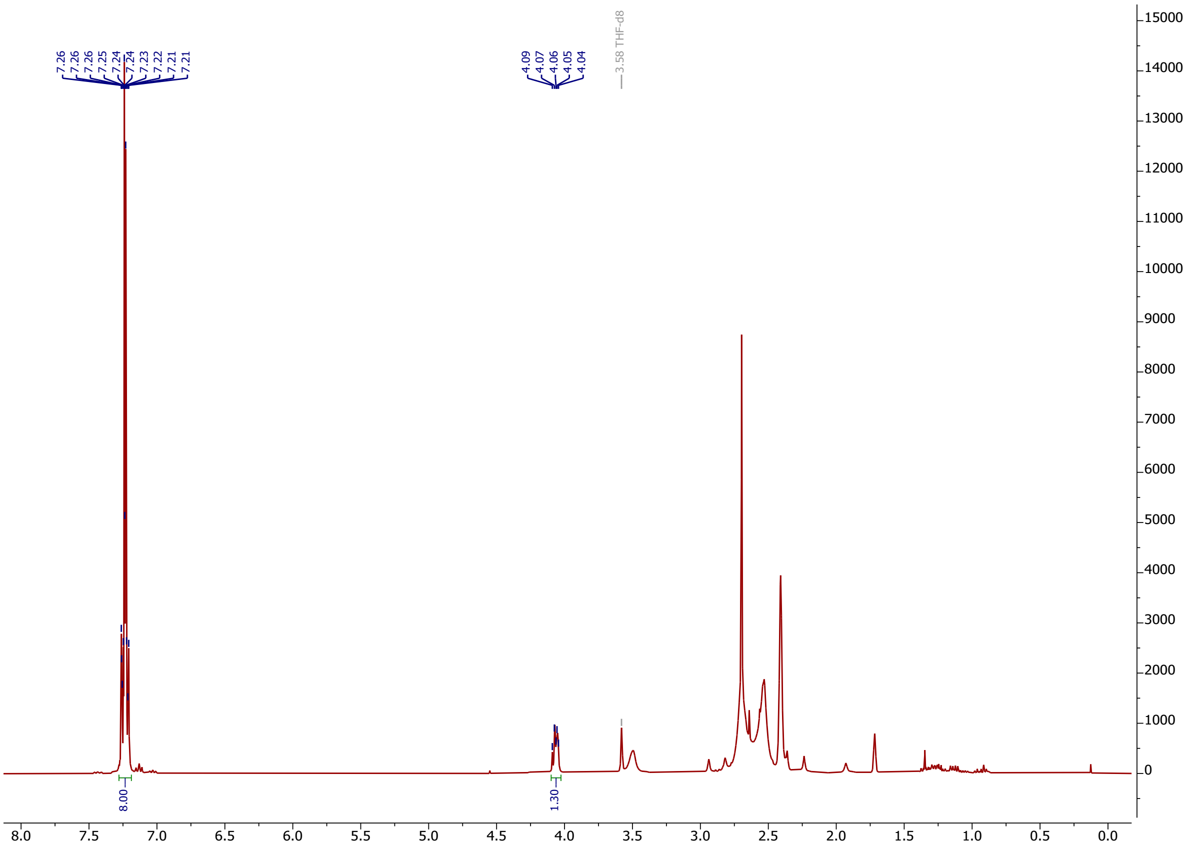

Figure S12: ^{1}H-NMR for reduction of 4-flurobenzonitrile with DMAB *B-D_3* in presence of **Mn1** after 6 h.

4.3 *In Operando* NMR Analysis

4.3.1 **Reduction of 4-fluorobenzonitrile**

Inside an argon-flushed glovebox, an NMR tube was charged with **Mn1** (0.0112 mmol, 2 mol%), 4-flurobenzonitrile (0.56 mmol, 1.0 equiv.), DMAB (0.73 mmol, 1.3 equiv.) and 0.56 mL C_6D_6 (1M). NMRs were periodically recorded.

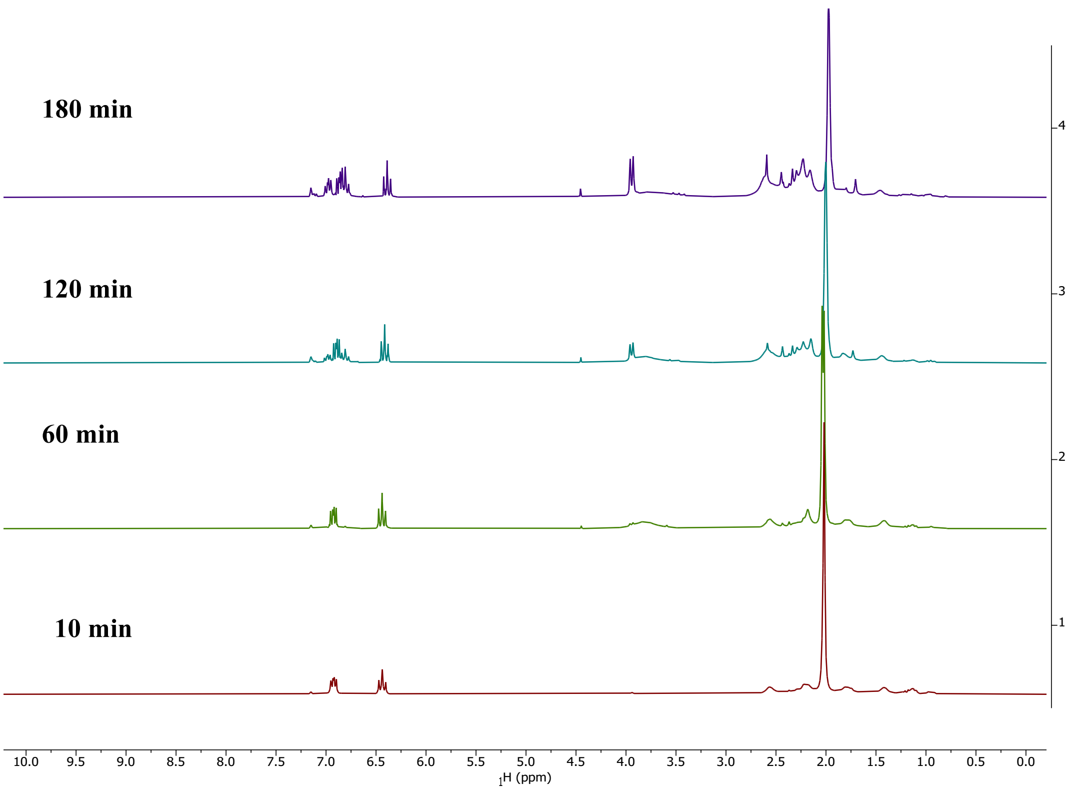

Figure S13: ^{1}H-NMR for reduction of 4-flurobenzonitrile with DMAB in presence of **Mn1** at different reaction times.

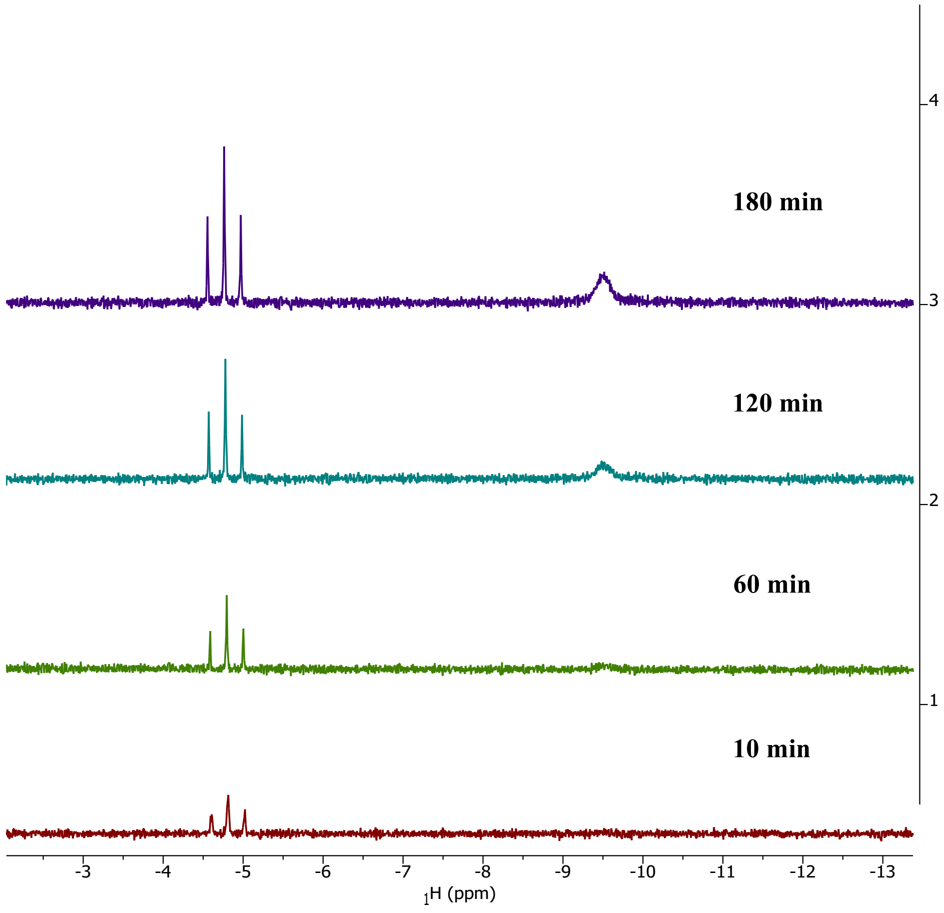

Figure S14: ^{1}H-NMR for reduction of 4-flurobenzonitrile with DMAB in presence of **Mn1** at different reaction times (hydridic region).

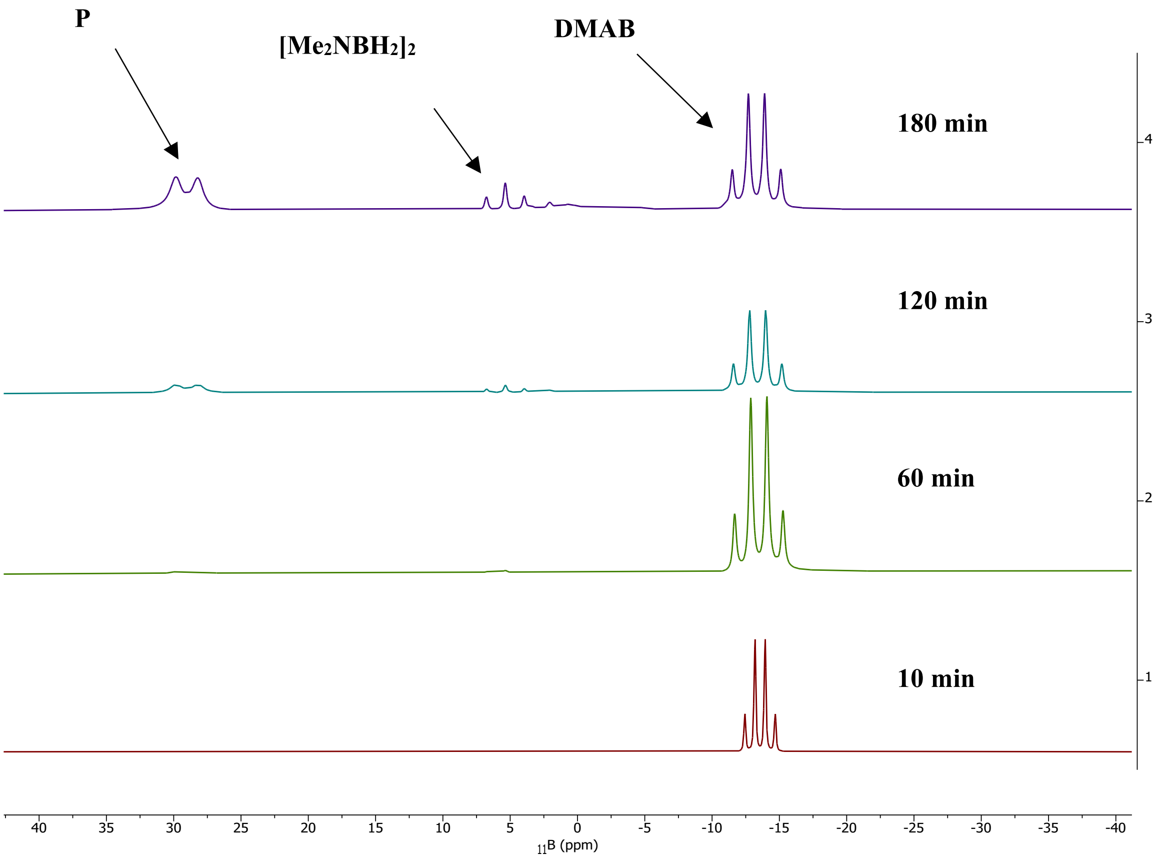

Figure S15: ^{11}B-NMR for reduction of 4-flurobenzonitrile with DMAB in presence of **Mn1** at different reaction times.

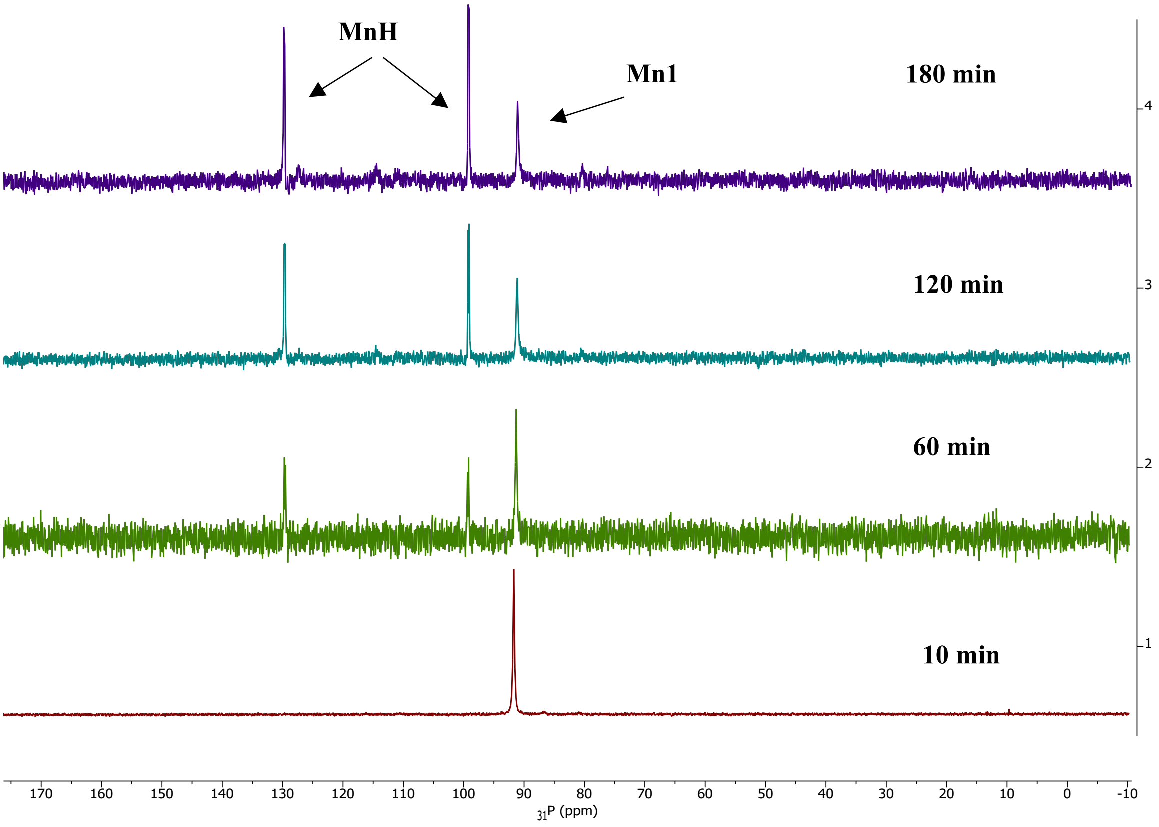

Figure S16: $^{31}P\{^{1}H\}$-NMR for reduction of 4-flurobenzonitrile with DMAB in presence of **Mn1** at different reaction times.

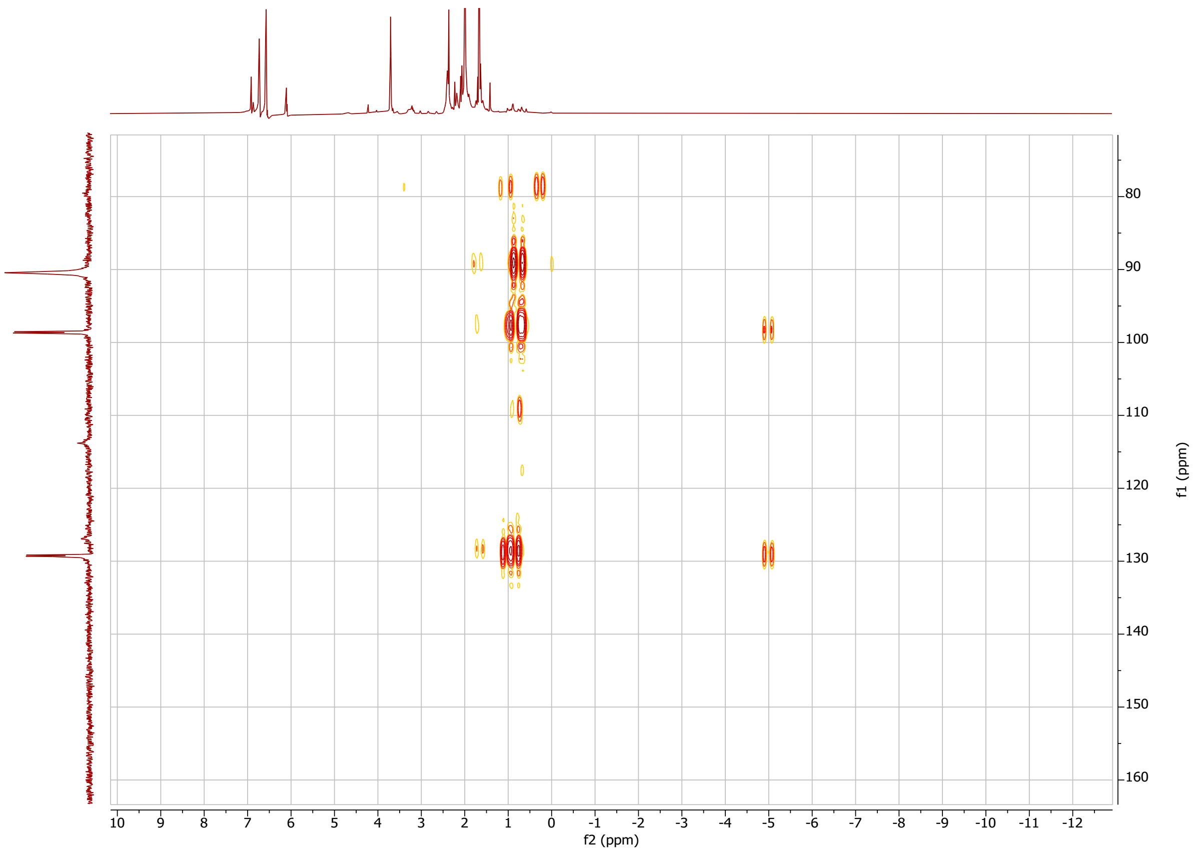

Figure S17: $^{1}H/^{31}P$-HMBC reduction of 4-flurobenzonitrile with DMAB in presence of **Mn1** at 90 min reaction time.

4.3.2 Reduction of various nitrile substrates

Inside an argon-flushed glovebox, an NMR tube was charged with **Mn1** (0.0112 mmol, 2 mol%), nitrile (0.56 mmol, 1.0 equiv.), DMAB (0.73 mmol, 1.3 equiv.) and 0.56 mL C_6D_6 (1M). NMRs were periodically recorded.

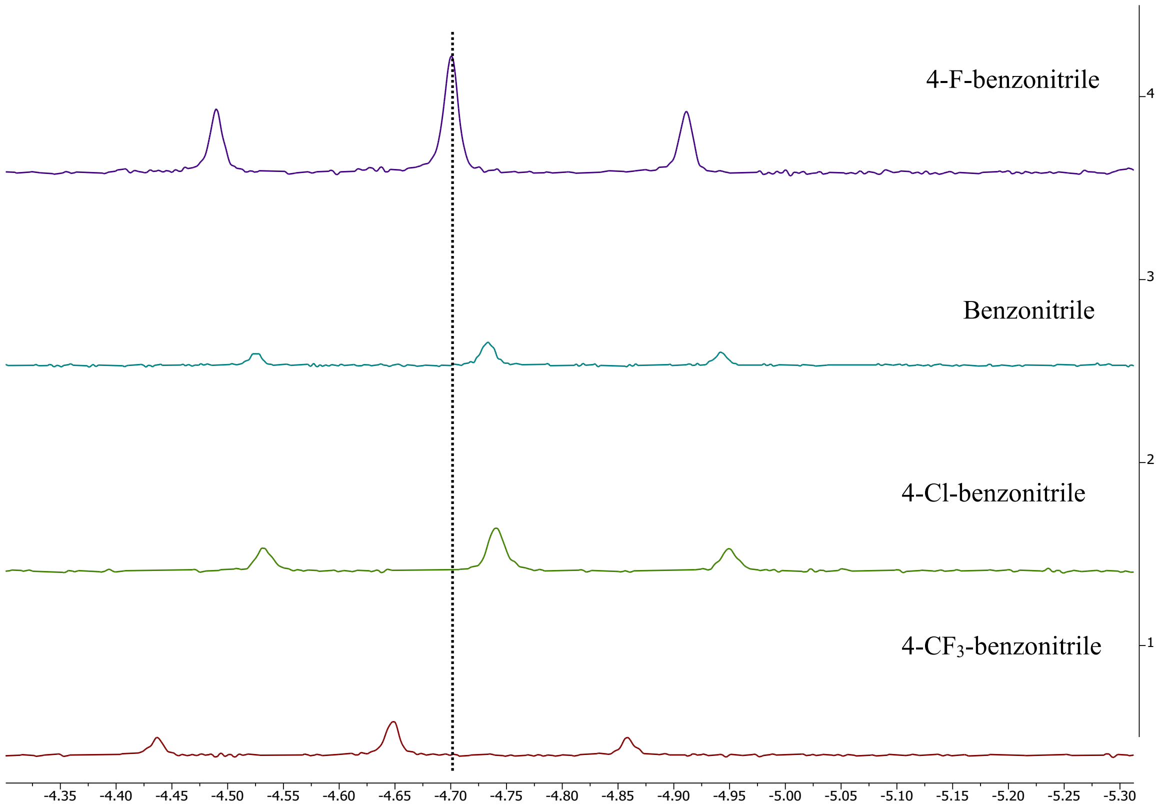

Figure S18: ^{1}H-NMR for reduction of nitriles with DMAB in presence of **Mn1** only hydridic region shown.

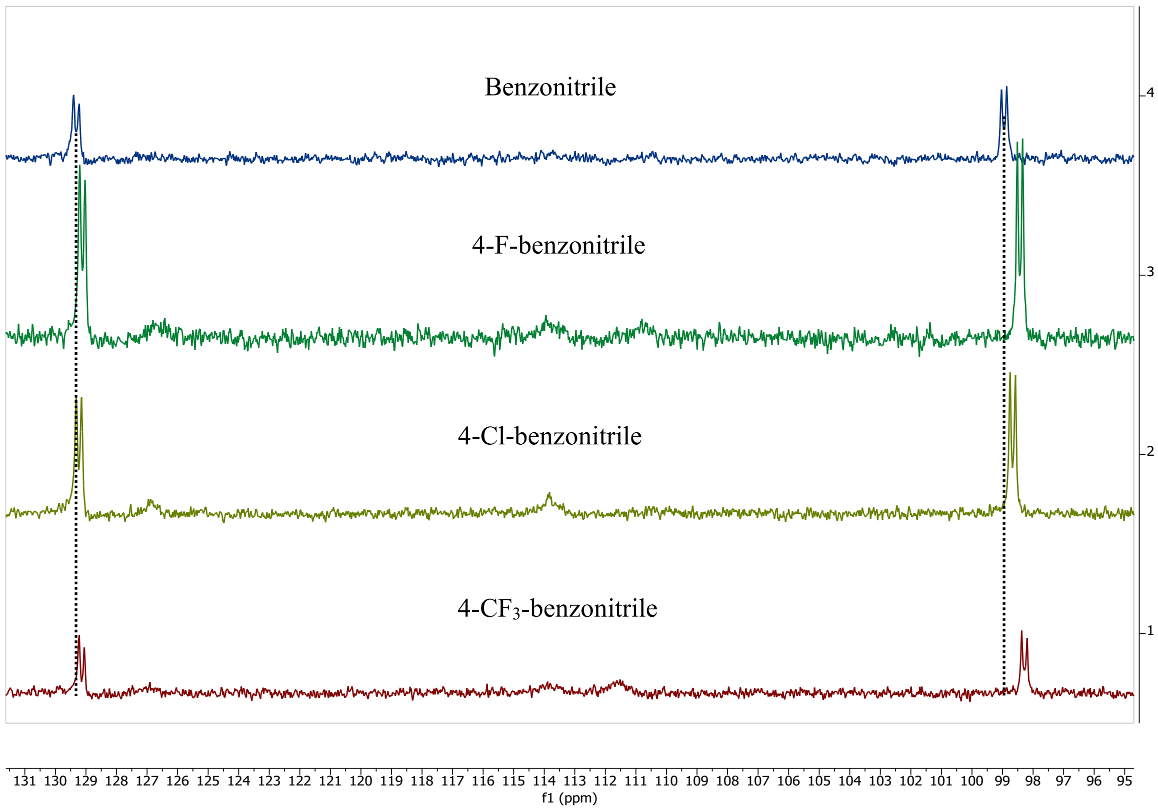

Figure S19: ^{31}P-NMR for reduction of nitriles with DMAB in presence of **Mn1.**

4.3.3 Reduction of 4-fluorobenzonitrile with DMAB *N-D* or B-D_3

Inside an argon-flushed glovebox, an NMR tube was charged with **Mn1** (0.0112 mmol, 2 mol%), 4-flurobenzonitrile (0.56 mmol, 1.0 equiv.), DMAB *N-D* or B-D_3 (0.73 mmol, 1.3 equiv.) and 0.56 mL C_6D_6 (1M). NMRs were periodically recorded.

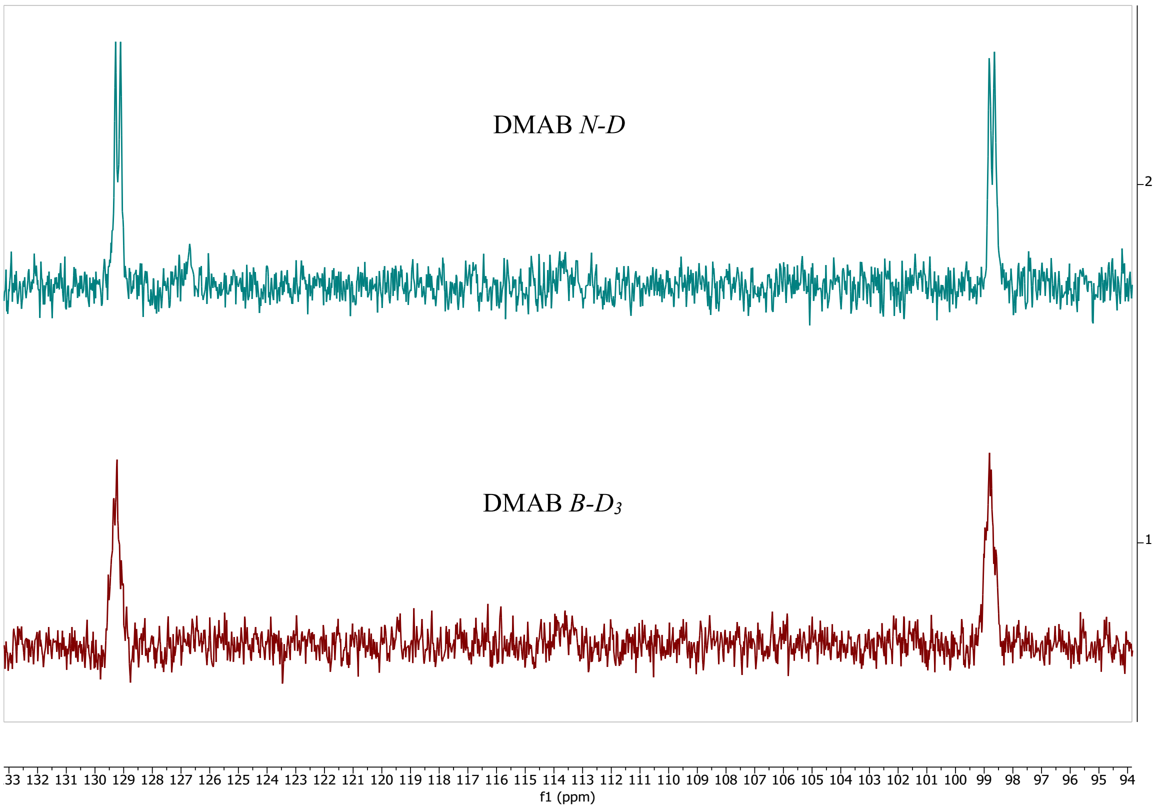

Figure S20: ^{31}P-NMR for reduction of 4-fluorobenzonitriles with DMAB N-*D* and B-D_3 in presence of **Mn1.**

4.4 Kinetic experiments

4.4.1 Variation of catalyst loading

Inside an argon-flushed glovebox, an NMR tube was charged with **Mn1** (1-3 mol%), 4-flurobenzonitrile (0.56 mmol, 1.0 equiv.), DMAB (0.73 mmol, 1.3 equiv.) and 0.56 mL C_6D_6 (1M). NMRs were periodically recorded.

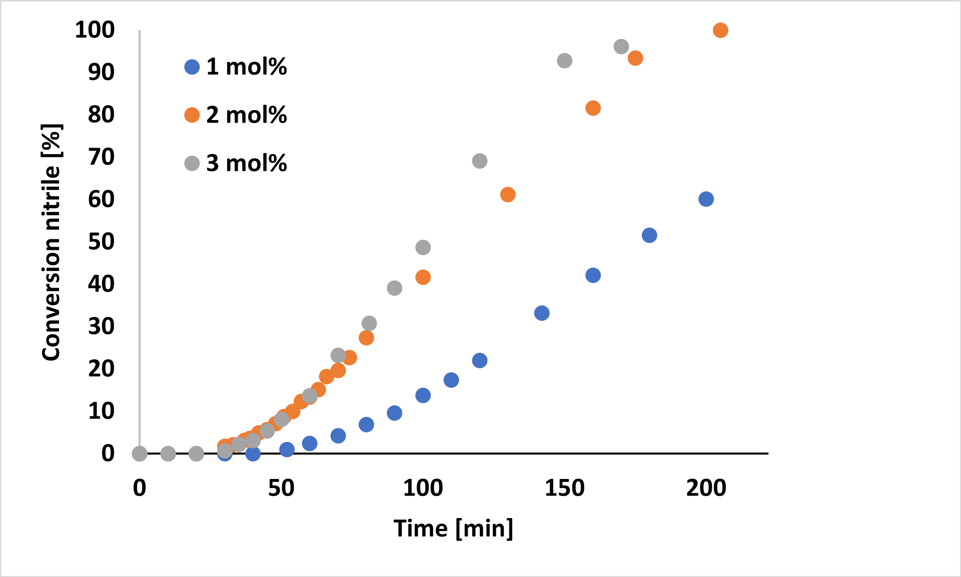

Figure S21: Kinetic profile of reduction of 4-flurobenzonitrile with various catalyst loadings.

4.4.2 Variation of substrate amount

Inside an argon-flushed glovebox, an NMR tube was charged with **Mn1** (0.0112 mmol, 2 mol%), 4-flurobenzonitrile (0.5- 1.0 equiv.), DMAB (0.73 mmol, 1.3 equiv.) and 0.56 mL C_6D_6 (1M). NMRs were periodically recorded.

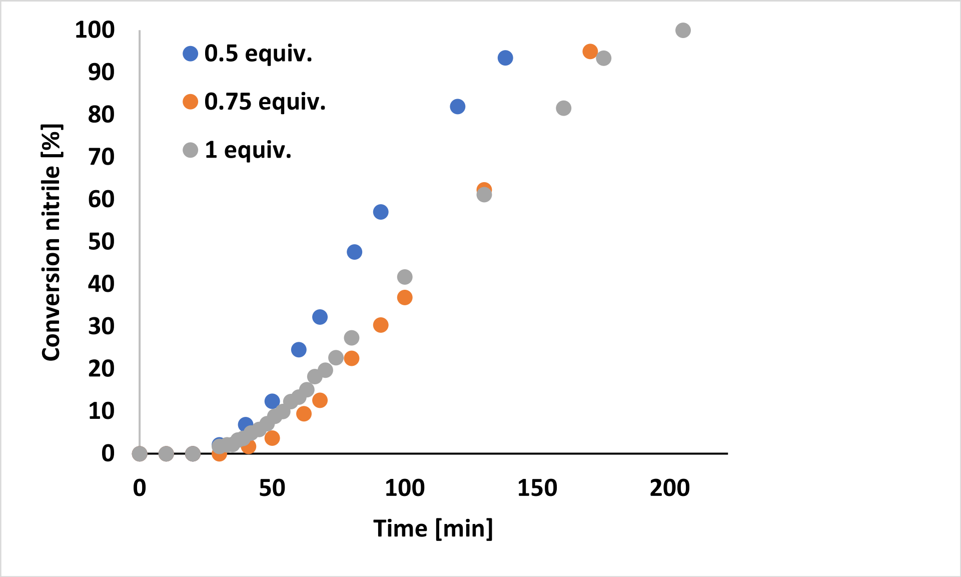

Figure S22: Kinetic profile of reduction of 4-flurobenzonitrile with various amount of substrate.

4.4.3 Variation of DMAB amount

Inside an argon-flushed glovebox, an NMR tube was charged with **Mn1** (0.0112 mmol, 2 mol%), 4-flurobenzonitrile (0.56 mmol, 1.0 equiv.), DMAB (0.73 mmol, 1.0-1.7 equiv.) and 0.56 mL C_6D_6 (1M). NMRs were periodically recorded.

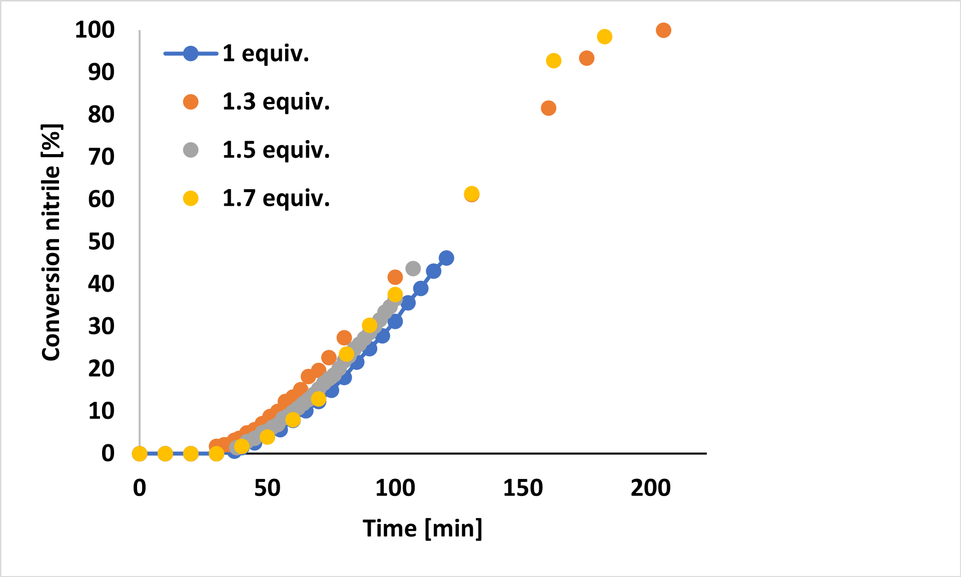

Figure S23: Kinetic profile of reduction of 4-flurobenzonitrile with various amount of DMAB.

4.4.4 Employing DMAB *N-D* or B-*D_3*

Inside an argon-flushed glovebox, an NMR tube was charged with **Mn1** (0.0112 mmol, 2 mol%), 4-flurobenzonitrile (0.56 mmol, 1.0 equiv.), DMAB *N-D* or B-*D_3* (0.73 mmol, 1.3 equiv.) and 0.56 mL C_6D_6 (1M). NMRs were periodically recorded.

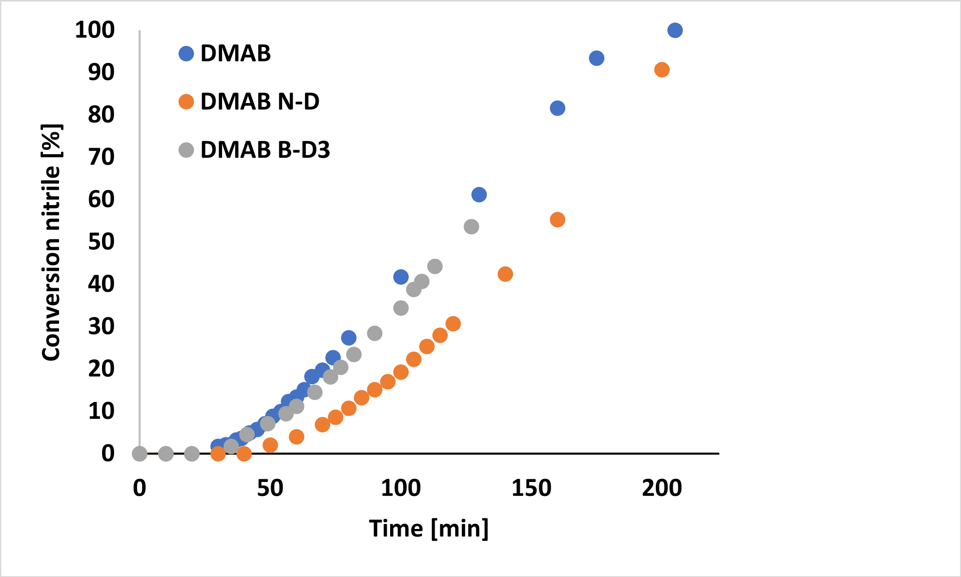

Figure S24: Kinetic profile of reduction of 4-flurobenzonitrile with DMAB *N-D* or B-*D_3*.

4.4.5 Employing various nitrile substrates

Inside an argon-flushed glovebox, an NMR tube was charged with **Mn1** (0.0112 mmol, 2 mol%), nitrile (0.56 mmol, 1.0 equiv.), DMAB (0.73 mmol, 1.3 equiv.) and 0.56 mL C_6D_6 (1M). NMRs were periodically recorded.

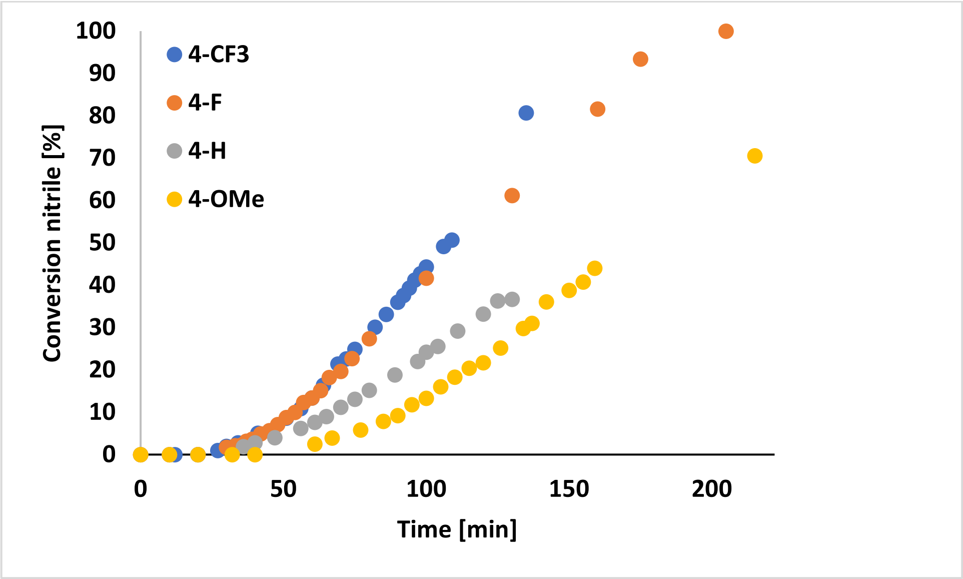

Figure S25: Kinetic profile of reduction of various *para*-substituted benzonitriles with DMAB.

5 Characterization of Organic Products

4-Flurobenzylammonium chloride[4] (**1**): pale yellow solid, ^{1}H NMR (δ, 400 MHz, D_2O, 20 °C): 7.37 – 7.30 (*m*, 2H), 7.04 (*m*, 2H), 4.03 (*s*, 2H). $^{13}C\{^1H\}$ NMR (δ, 101 MHz, D_2O, 20 °C):162.4, 131.0 , 128.6 , 115.8 , 42.4 .

Benzylammonium chloride[4] (**3**): white solid, ^{1}H NMR (δ, 400 MHz, CD_3OD, 20 °C): 7.33 – 7.18 (*m*, 5H), 3.79 (*s*, 2H). $^{13}C\{^1H\}$ NMR (δ, 101 MHz, CD_3OD, 20 °C):142.3, 128.1 , 127.0 , 126.5, 45.3.

4-(Trifluoromethyl)benzylammonium chloride[5] (**5**): white solid, ^{1}H NMR (δ, 250 MHz, D_2O, 20 °C): 7.68 (*d*, J = 8.1 Hz, 2H), 7.50 (*d*, J = 8.1 Hz, 2H), 4.16 (*s*, 2H). $^{13}C\{^1H\}$ NMR (δ, 63 MHz, D_2O, 20 °C):136.5 , 130.3, 139.2 , 126.0 (q, J= , 42.9) .

4-(Methyl)benzylammonium chloride[6] (**6**): white solid, ^{1}H NMR (δ, 400 MHz, D_2O, 20 °C): 7.35-7.29 (m, 4H), 4.07 (s, 2H), 2.34 (s, 3H). $^{13}C\{^1H\}$ NMR (δ, 100 MHz, D_2O, 20 °C): 139.2, 131.0, 129.7, 128.6, 43.1, 20.2.

Methyl (4-methyammoniun)benzoate chloride[4] (7): white solid, ^{1}H NMR (400 MHz, D_2O) δ = 7.34 (d, *J* = 8.2 Hz, 2H), 6.95 (d, *J* = 8.1 Hz, 2H), 4.05 (s, 2H), 3.34 (s, 3H). $^{13}C\{^1H\}$ NMR (100 MHz, D_2O) δ = 159.3, 130.6. 125.1, 114.5, 55.3, 42.1.

4-(Methoxy)benzylammonium chloride[6] (**8**): pale yellow solid, ^{1}H NMR (δ, 400 MHz, D_2O, 20 °C): 8.02 (d, *J* = 8.0 Hz, 2H), 7.54 (d, *J* = 7.9 Hz, 2H),4.26 (s, 2H), 3.11 (s, 3H). $^{13}C\{^1H\}$ NMR (δ, 100 MHz, D_2O, 20 °C): 169.7, 137.9, 130.0, 128.8, 52.7, 42.1.

(Napht-2-yl)methylammonium chloride[6] (**10**): white solid, ^{1}H NMR (δ, 400 MHz, CD_3OD, 20 °C): 7.50 – 7.48 (*m*, 4H), 7.42 – 7.34 (*m*, 4H), 4.12 (*s*, 2H). $^{13}C\{^1H\}$ NMR (δ, 101 MHz, CD_3OD, 20 °C):133.4, 133.3, 130.4, 128.7, 128.2, 127.7, 127.4, 126.5, 126.4, 125.7, 47.1.

(Thiophen-2yl)methylammonium chloride[7] (12): yellow solid, ^{1}H NMR (δ, 400 MHz, D_2O, 20 °C): 7.50-7.48 (m, 1H), 7.22-7.20 (m, 1H), 7.07-7.06 (m, 1H), 4.38 (s, 2H). $^{13}C\{^1H\}$ NMR (δ, 100 MHz, D_2O, 20 °C): 133.7, 129.5, 127.9, 127.7, 37.3.

(5-(4-chlorophenyl)furan-2-yl)methylammonium chloride[8] (13) yellow solid, ^{1}H NMR (400 MHz, MeOD) δ 7.72 (d, *J* = 8.5 Hz, 2H), 7.40 (d, *J* = 8.6 Hz, 2H), 6.83 (d, *J* = 3.3 Hz, 1H), 6.66 (d, *J* = 3.4 Hz, 1H), 4.24 (s, 2H). $^{13}C\{^1H\}$ NMR (δ, 100 MHz, D_2O, 20 °C): 154.1, 146.5, 133.3, 128.8, 128.6, 125.0, 112.8, 106.5, 35.6.

(Phenyl)ethylammonium chloride[6] (**15**): white solid ^{1}H NMR (400 MHz, D_2O) δ 7.36 (t, *J* = 7.3 Hz, 2H), 7.28 (d, *J* = 7.7 Hz, 3H), 3.20 (t, *J* = 7.2 Hz, 2H), 2.93 (t, *J* = 7.3 Hz, 2H). $^{13}C\{^1H\}$ NMR (δ, 100 MHz, D_2O, 20 °C): 137.0, 129.0, 128.9, 127,9, 40.6, 32.7.

1,10-Decandiammonium dichloride[5] (**15**): white solid, ^{1}H NMR (δ, 400 MHz, D_2O, 20 °C): 2.88 (*t*, J = 7.4 Hz, 4H), 1.63 – 1.46 (*m*, 8H), 1.27 (*m*, 20H). $^{13}C\{^1H\}$ NMR (δ, 100 MHz, D_2O, 20 °C): 39.5, 28.17, 3.82, 27.7, 25.5, 16.3.

(3-((Trimethylsilyl)ethynyl)phenyl)methylammonium chloride[4] (**17**): yellow solid, ^{1}H NMR (400 MHz, MeOD) δ 7.52 (s, 1H), 7.46 – 7.35 (m, 3H), 4.06 (s, 2H), 0.19 (s, 8H). ^{13}C NMR (100 MHz, MeOD) δ 131.9, 131.8, 129.0, 128.8, 124.0, 104., 94.2, 42.6, -1.5.

(*E*)-3-phenylprop-2-en-1-ammonium chloride[4] (18): white solid, ^{1}H NMR (400 MHz, MeOD) δ 7.44 (d, *J* = 7.4 Hz, 2H), 7.29 (dt, *J* = 23.5, 7.2 Hz, 3H), 6.79 (d, *J* = 15.7 Hz, 1H), 6.35 – 6.20 (m, 1H), 3.70 (d, *J* = 6.4 Hz, 2H). ^{13}C NMR (101 MHz, MeOD) δ 136.4, 135.7, 128.4, 128.2, 126.4, 119.8, 41.2.

6 NMR Spectra of Products

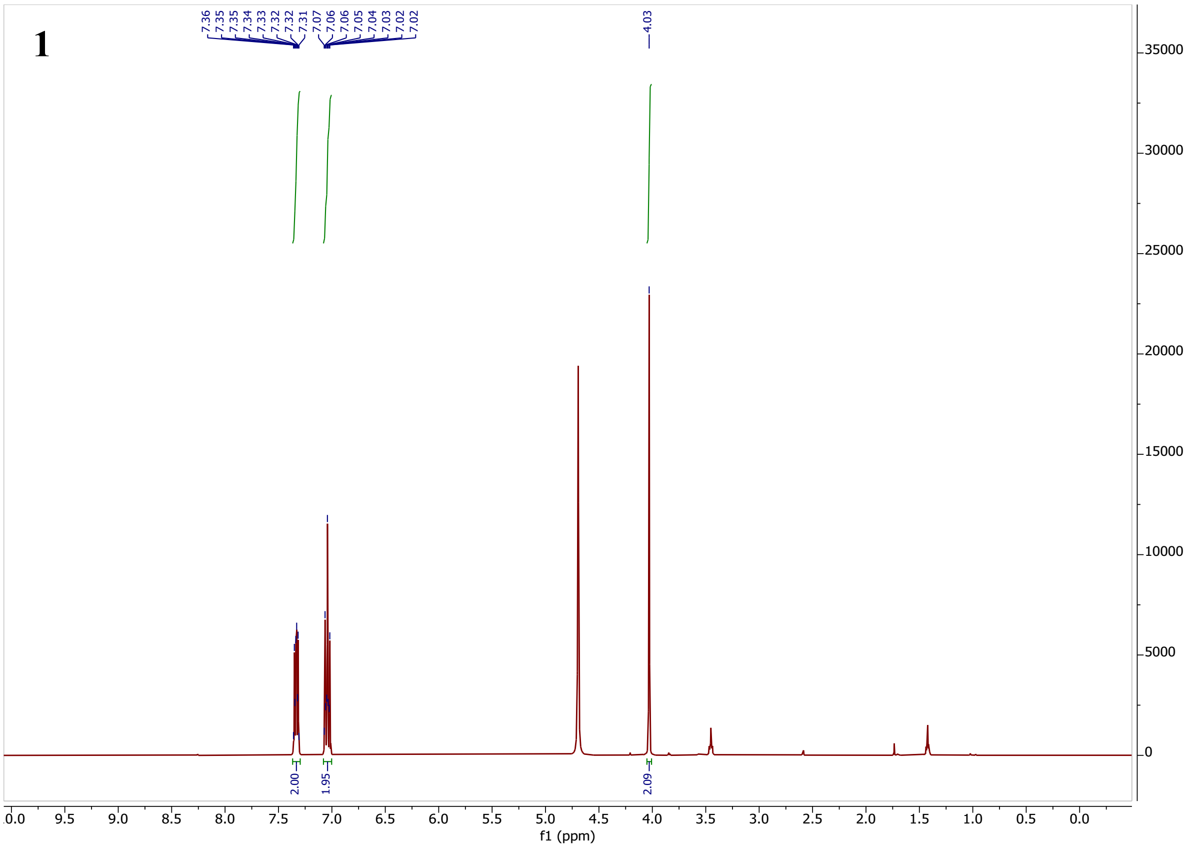

Figure S26. ^{1}H-NMR of isolated **1**.

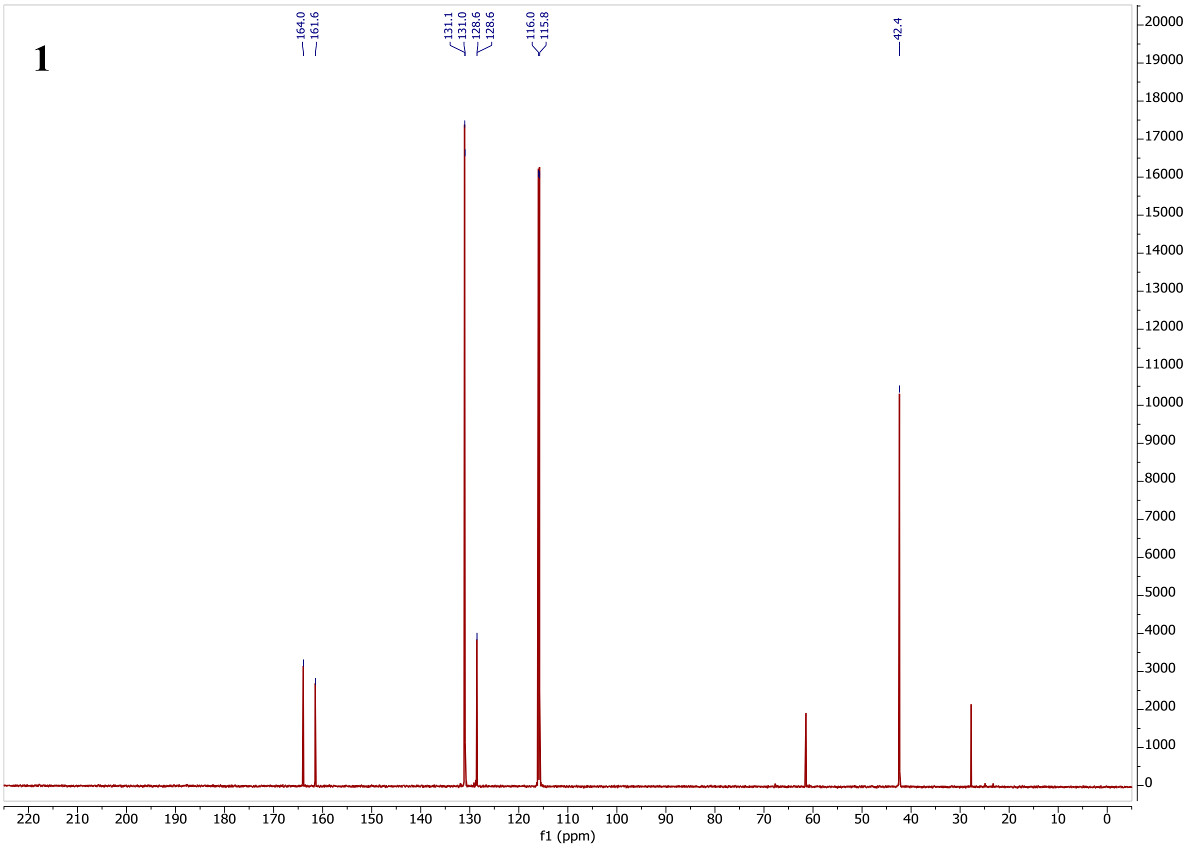

Figure S27. $^{13}C\{^{1}H\}$-NMR of isolated **1**.

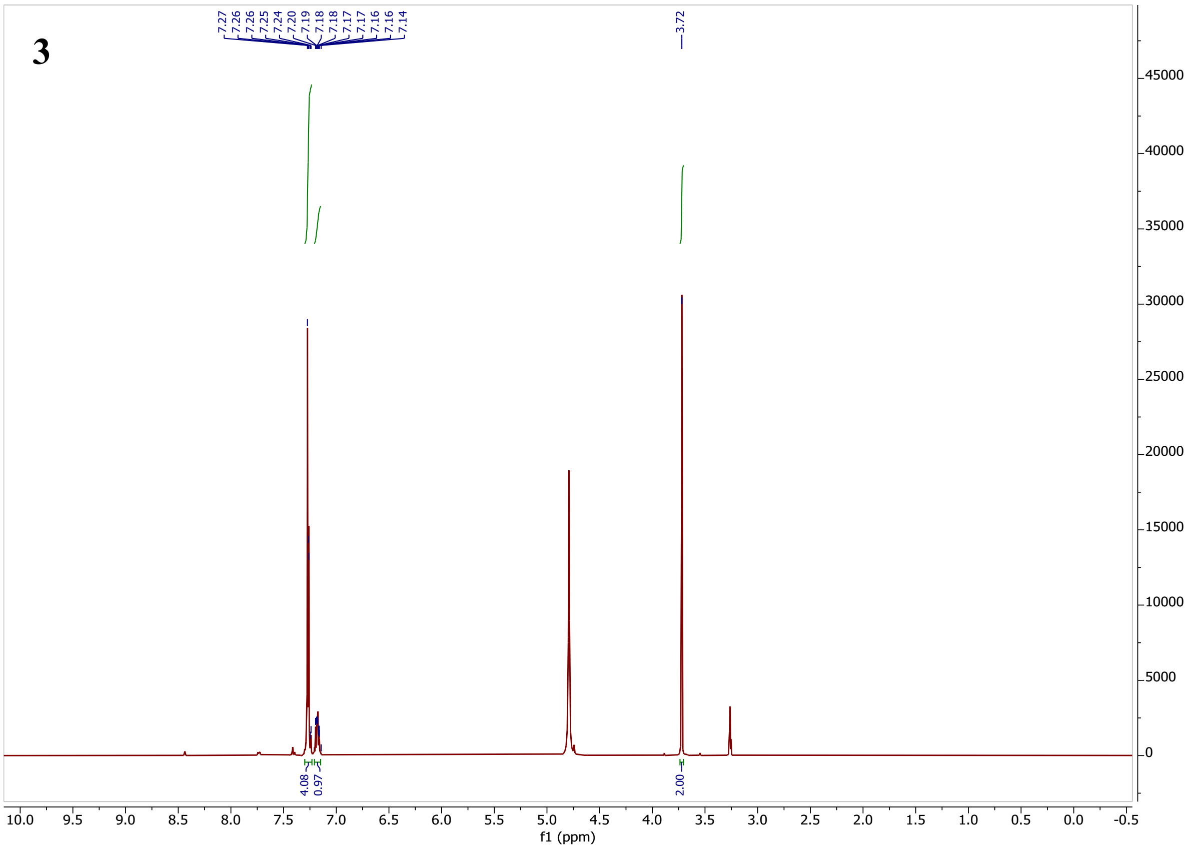

Figure S28. ^{1}H-NMR of isolated **3**.

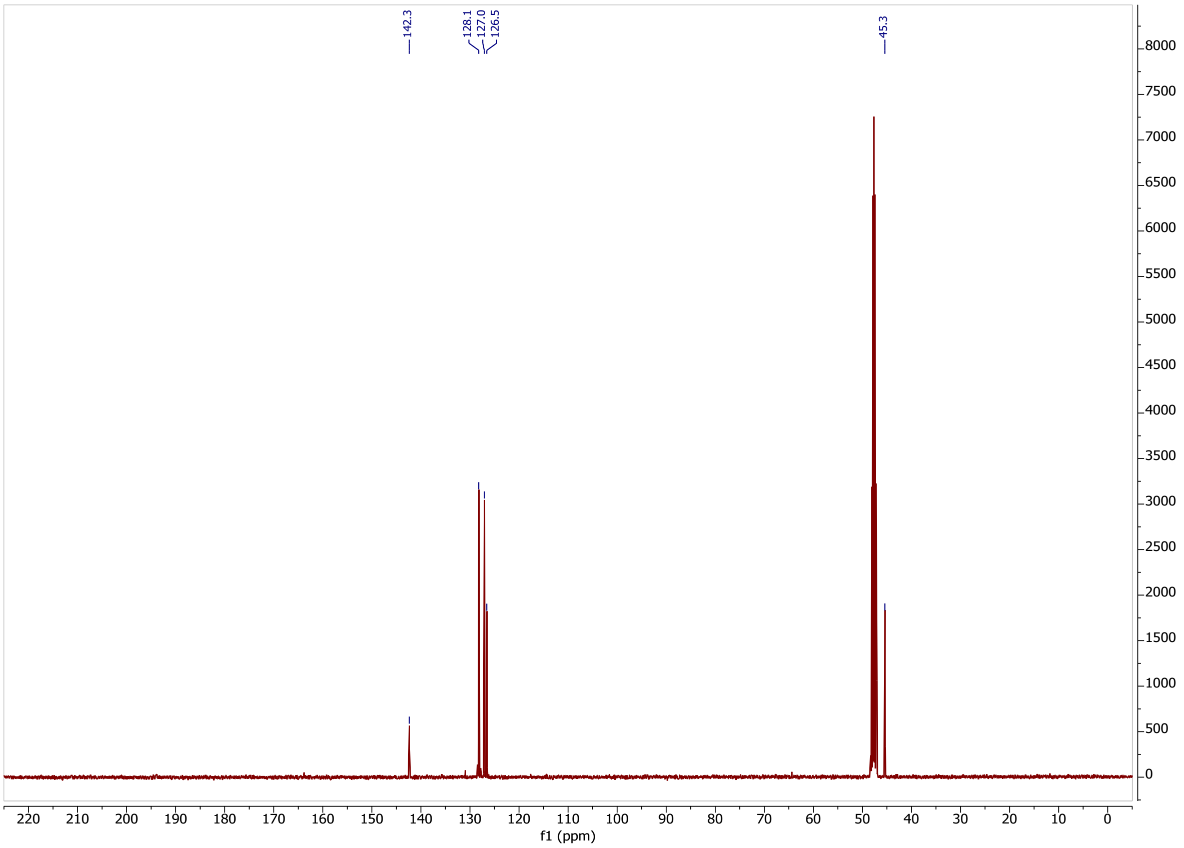

Figure S29. $^{13}C\{^{1}H\}$-NMR of isolated **3.**

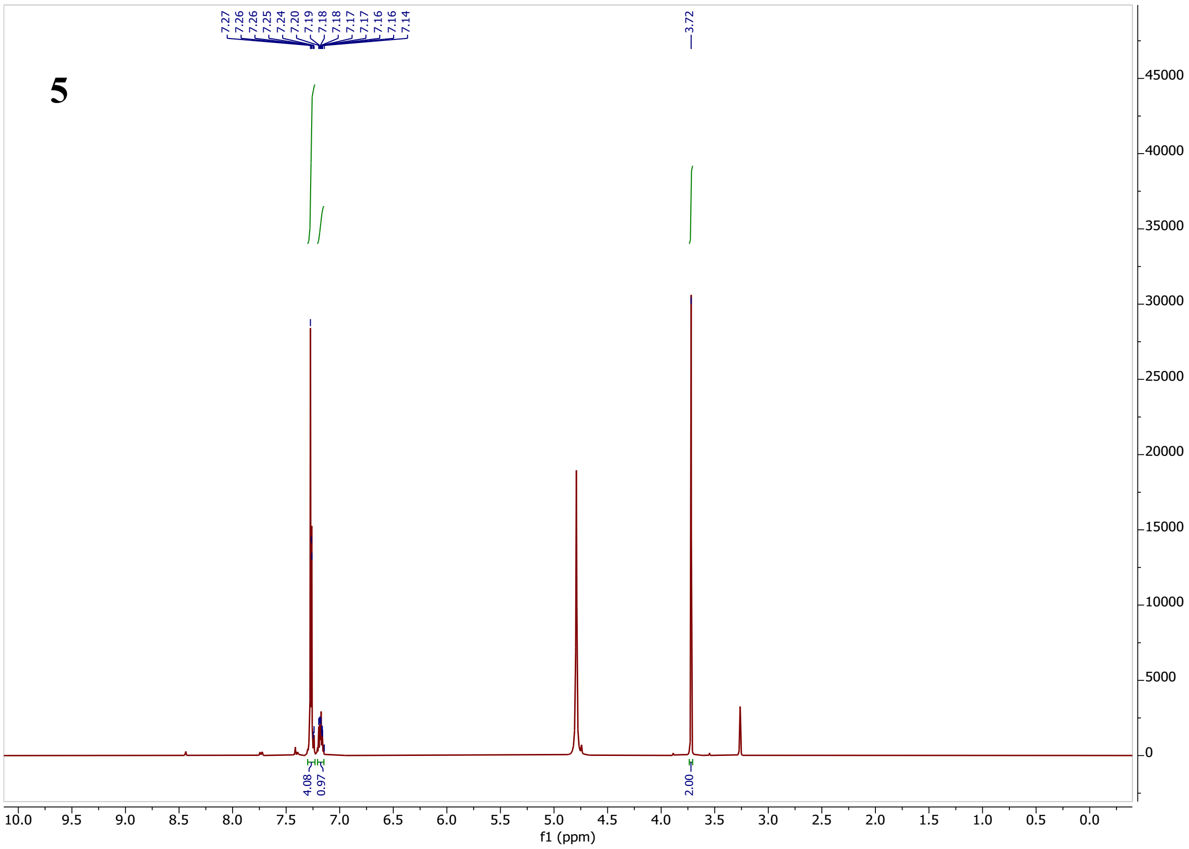

Figure S30. ^{1}H-NMR of isolated **5**.

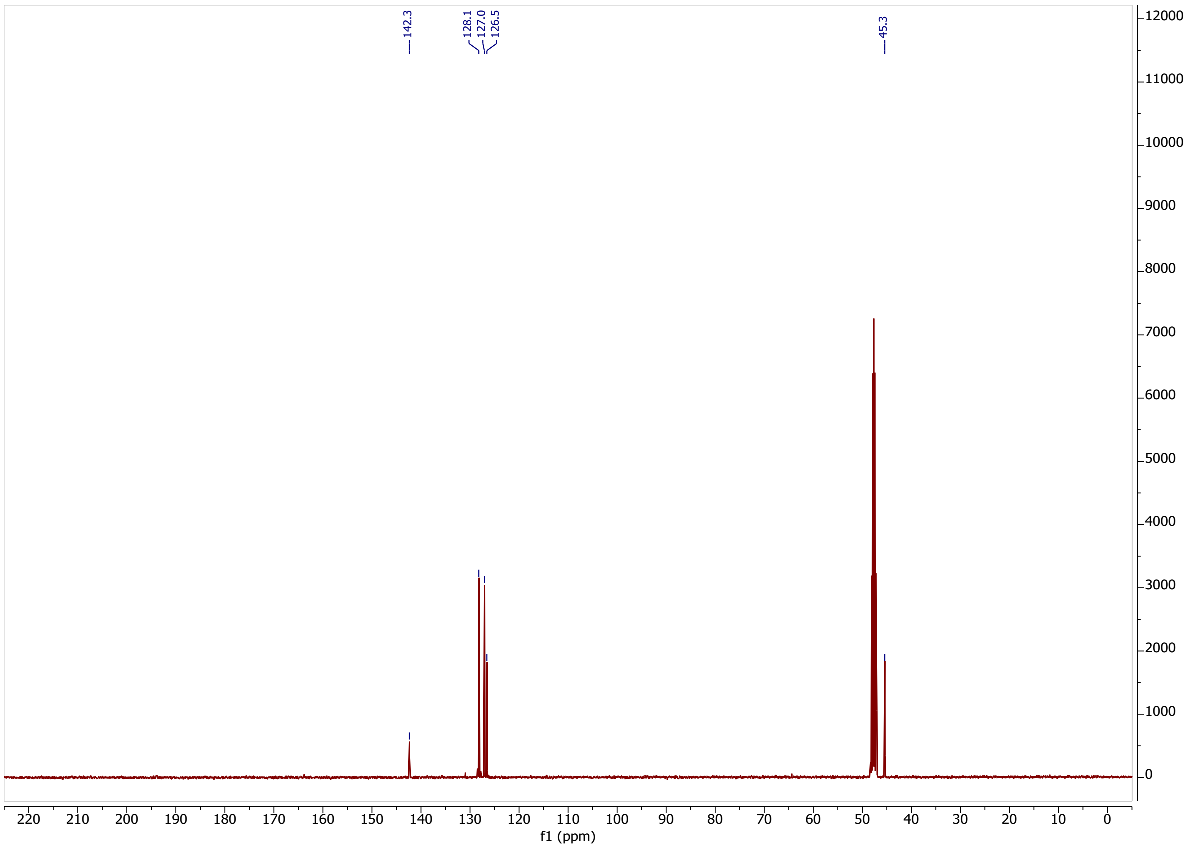

Figure S31. ^{13}C{^{1}H}-NMR of isolated **5.**

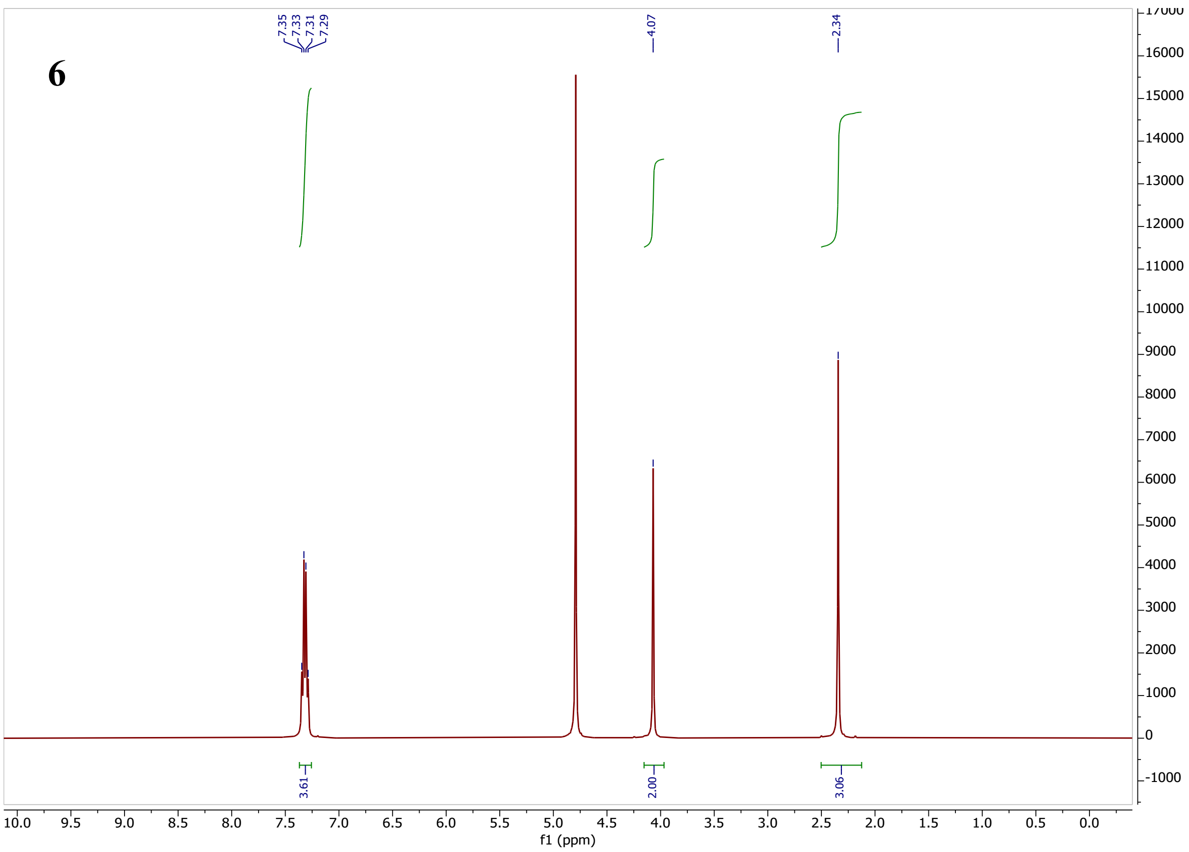

Figure S32. ^{1}H-NMR of isolated **6**.

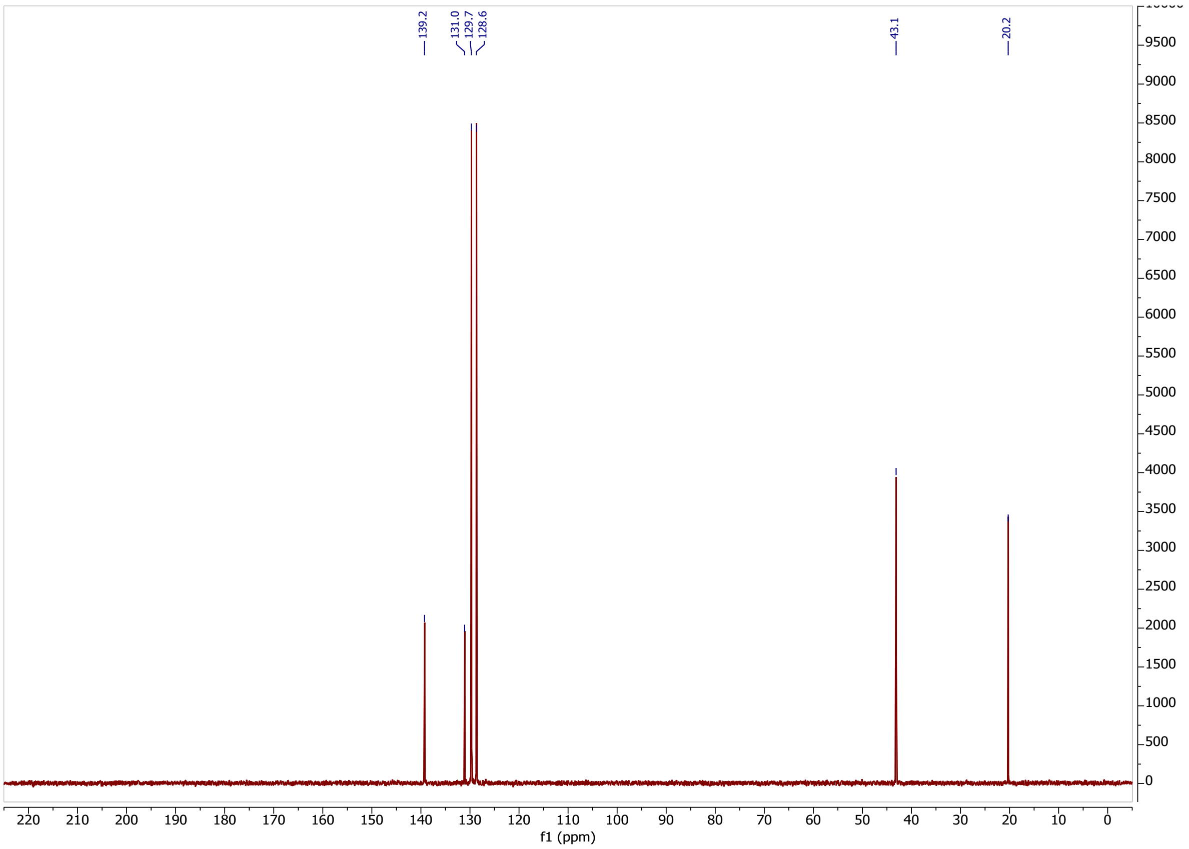

Figure S33. $^{13}C\{^{1}H\}$-NMR of isolated **6.**

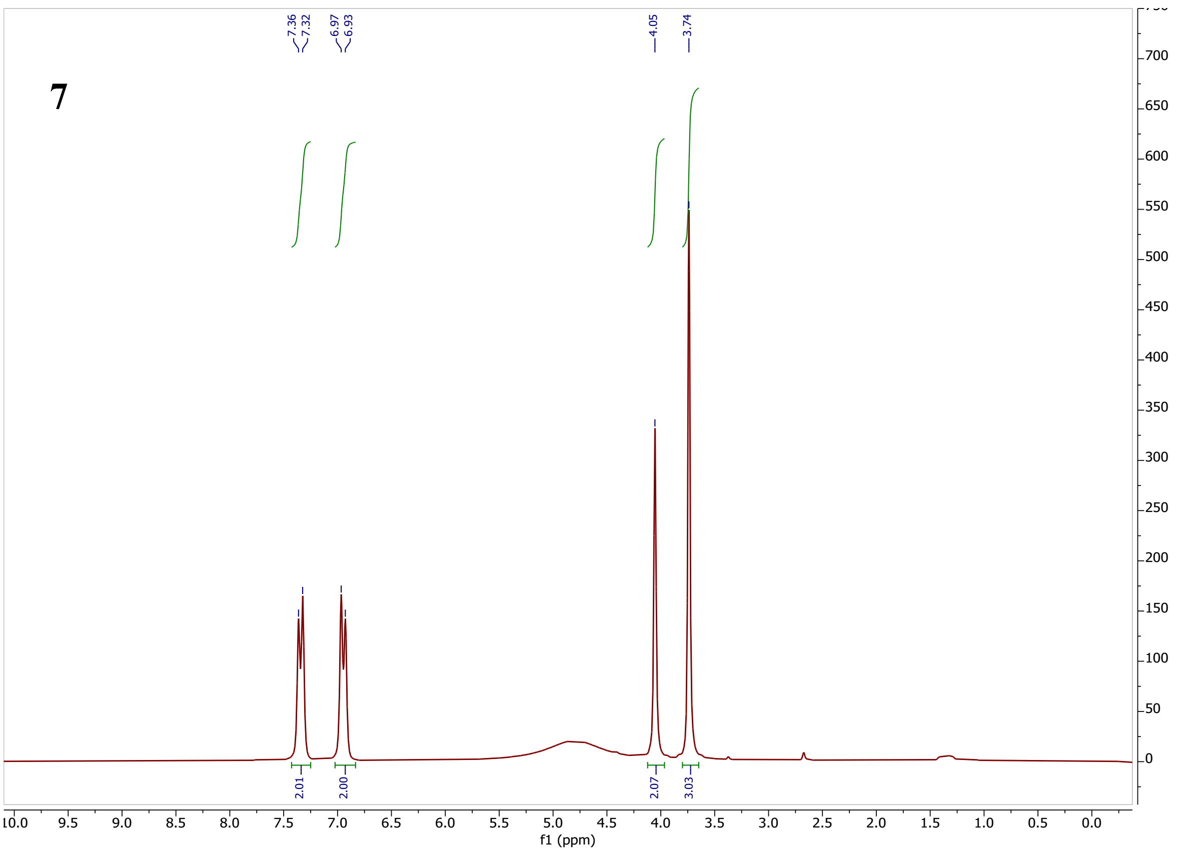

Figure S34. ^{1}H-NMR of isolated **7**.

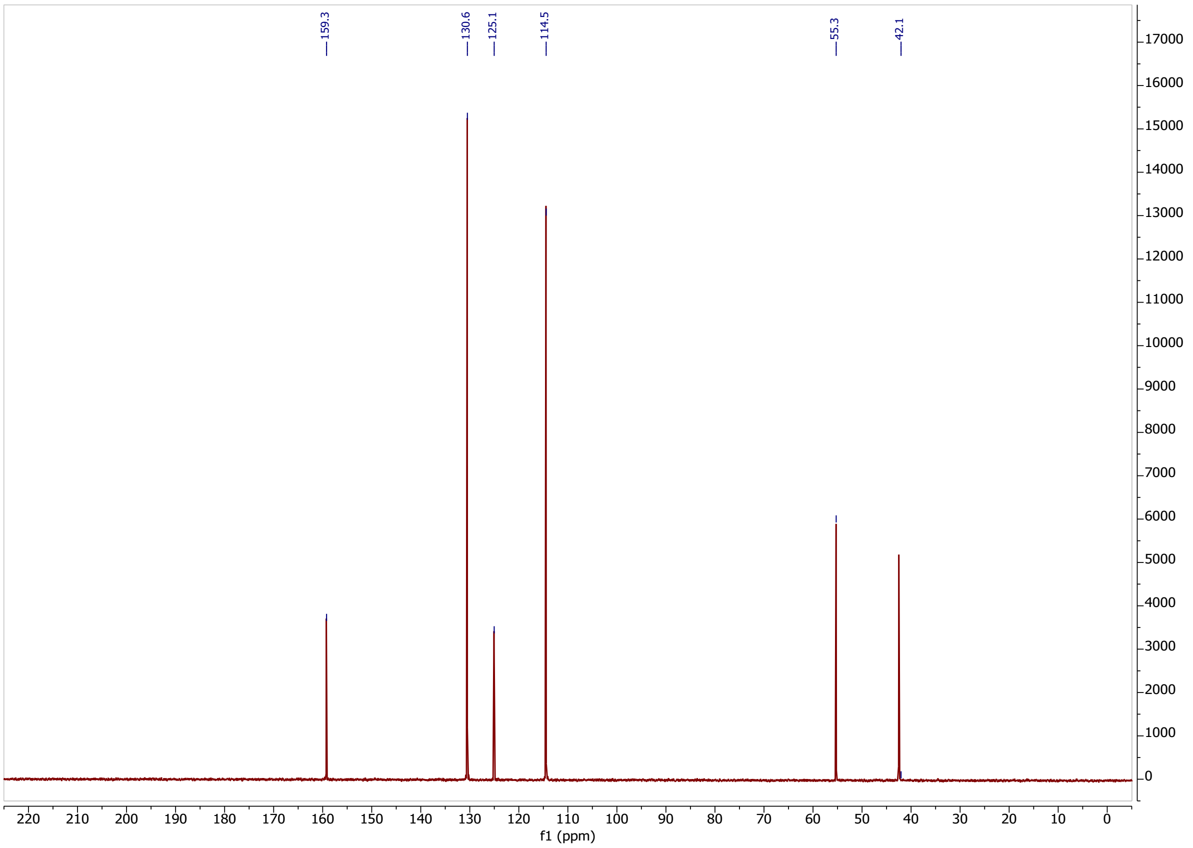

Figure S35. ^{13}C{^{1}H}-NMR of isolated **7.**

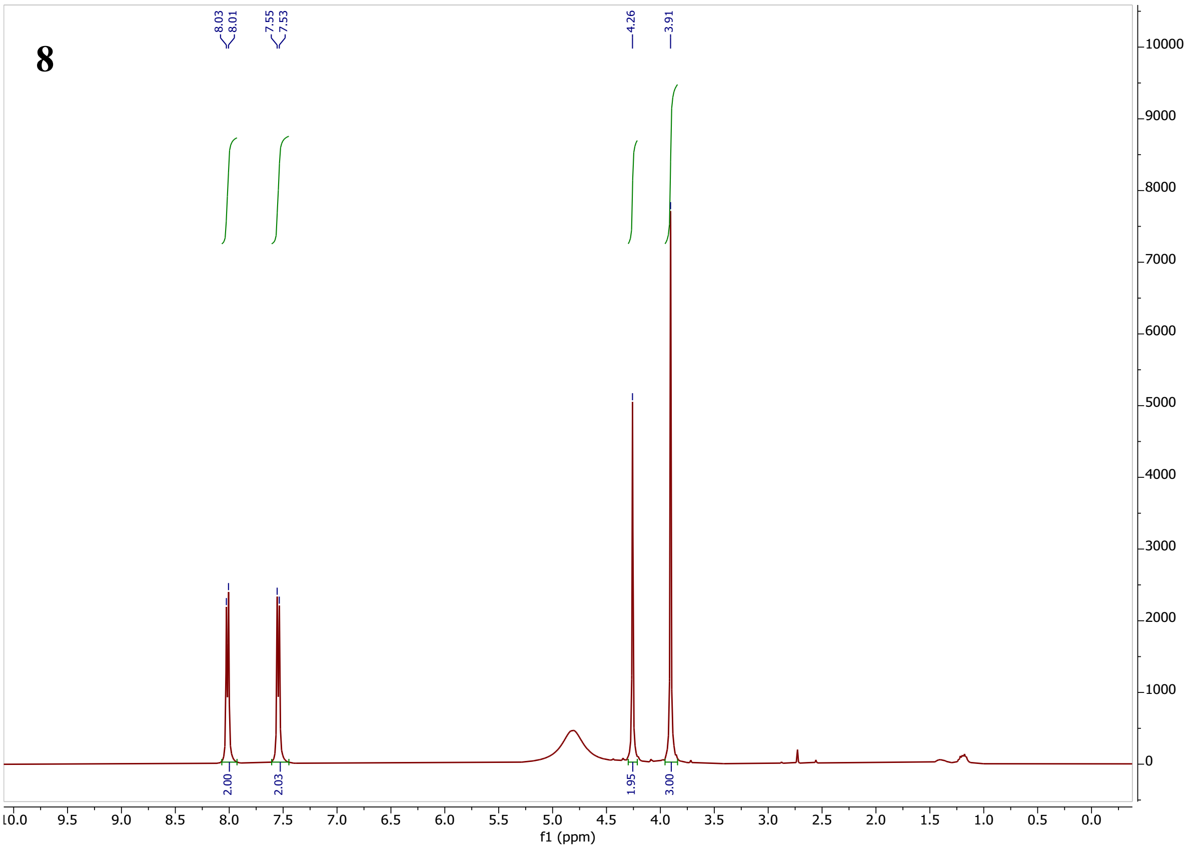

Figure S36. ^{1}H-NMR of isolated **8**.

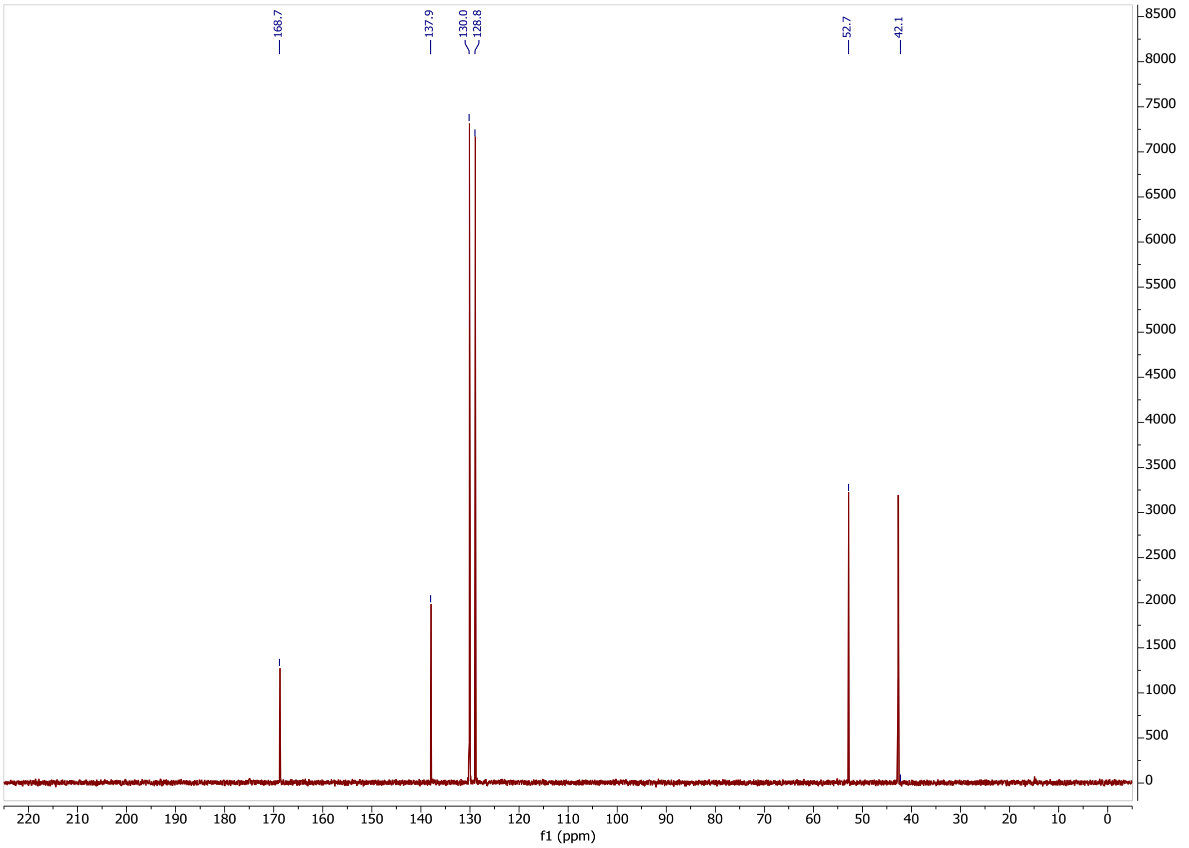

Figure S37. ^{13}C{^{1}H}-NMR of isolated **8.**

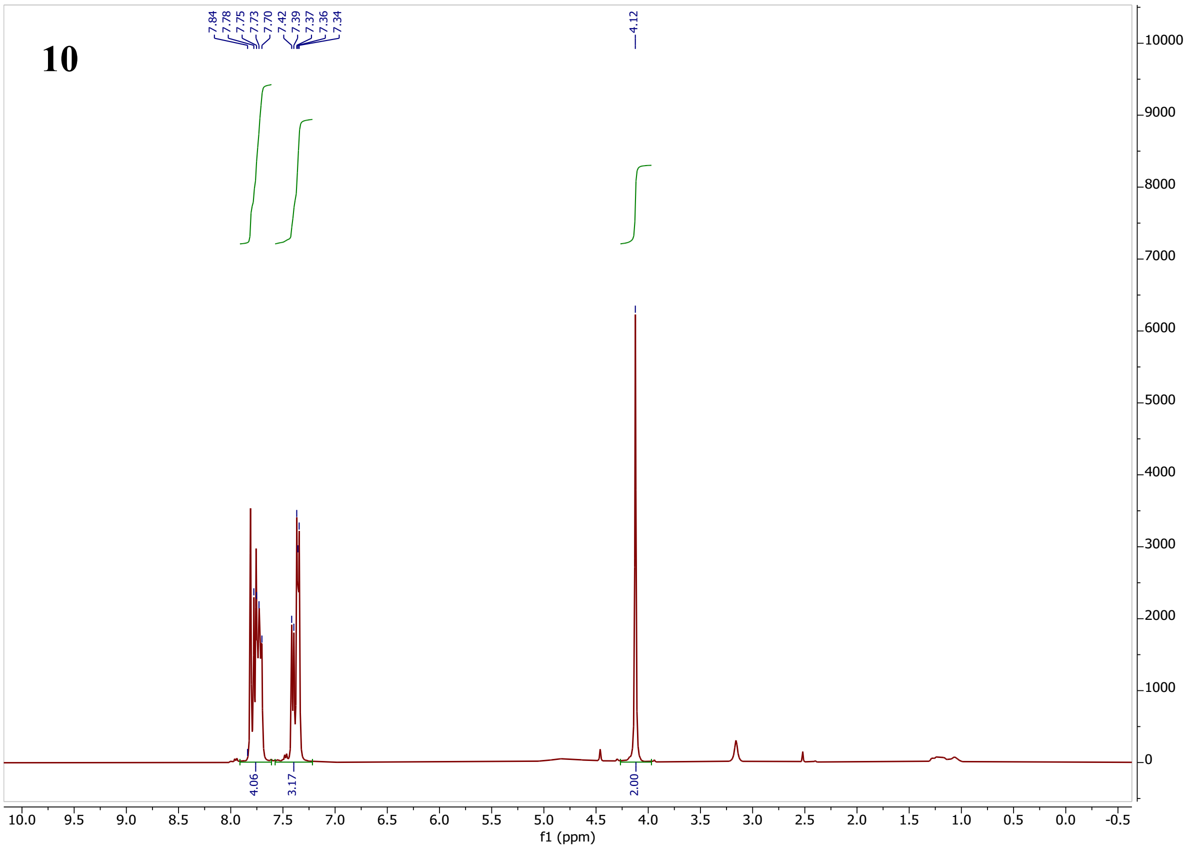

Figure S38. ^{1}H-NMR of isolated **10**.

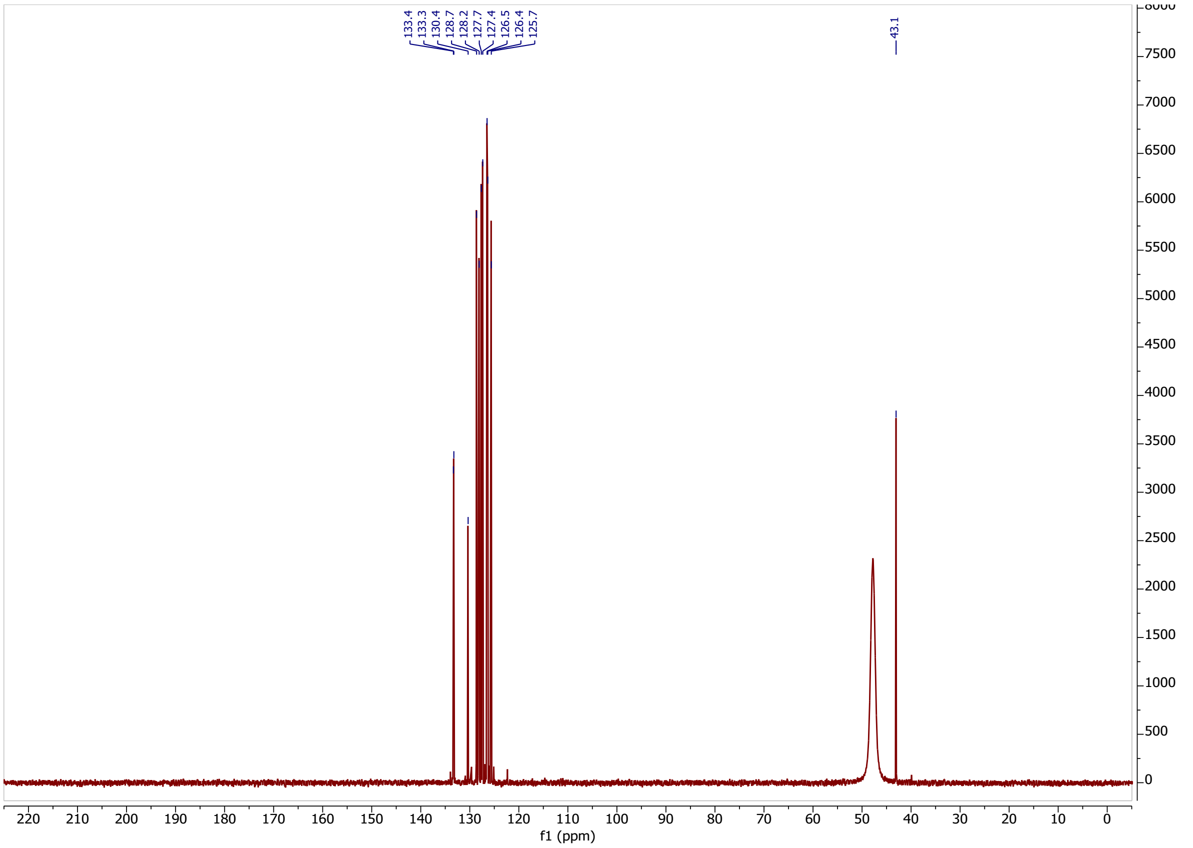

Figure S39. $^{13}C\{^{1}H\}$-NMR of isolated **10.**

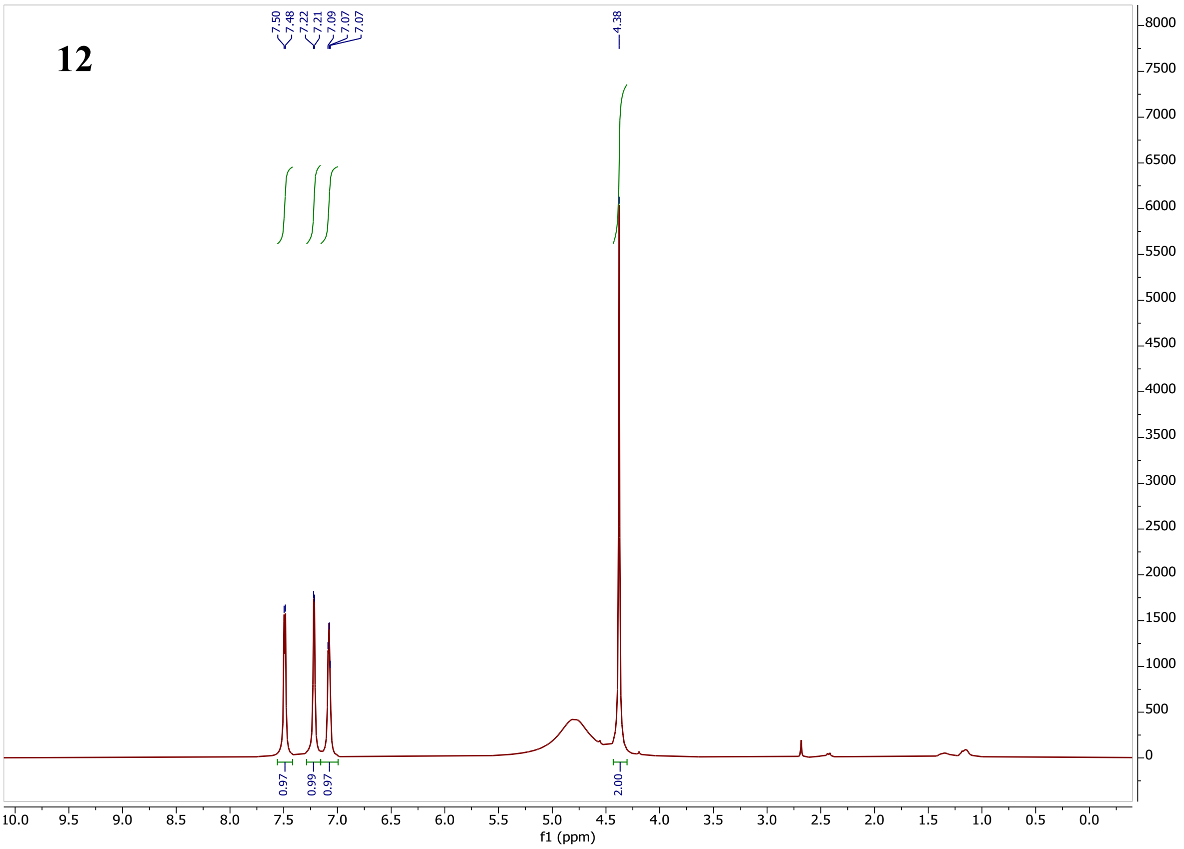

Figure S40. ^{1}H-NMR of isolated **12**.

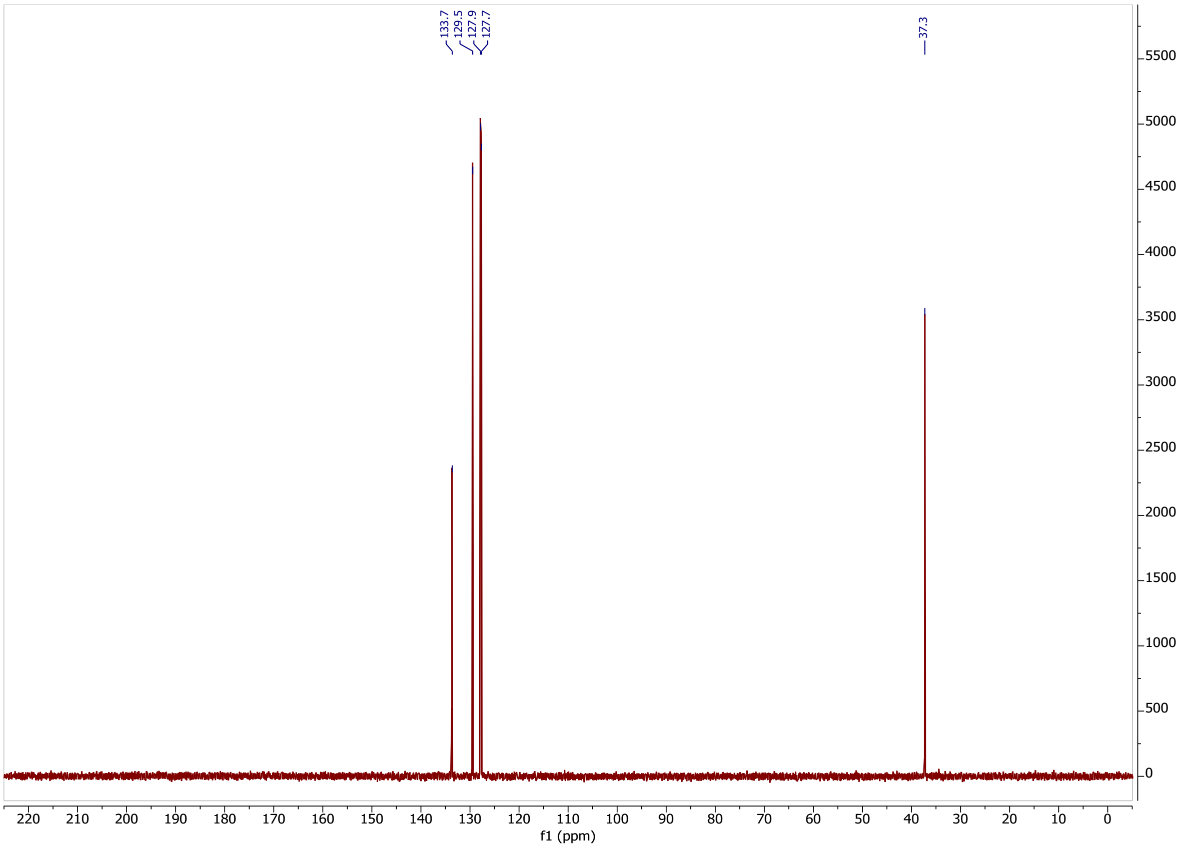

Figure S41. ^{13}C{^{1}H}-NMR of isolated **12.**

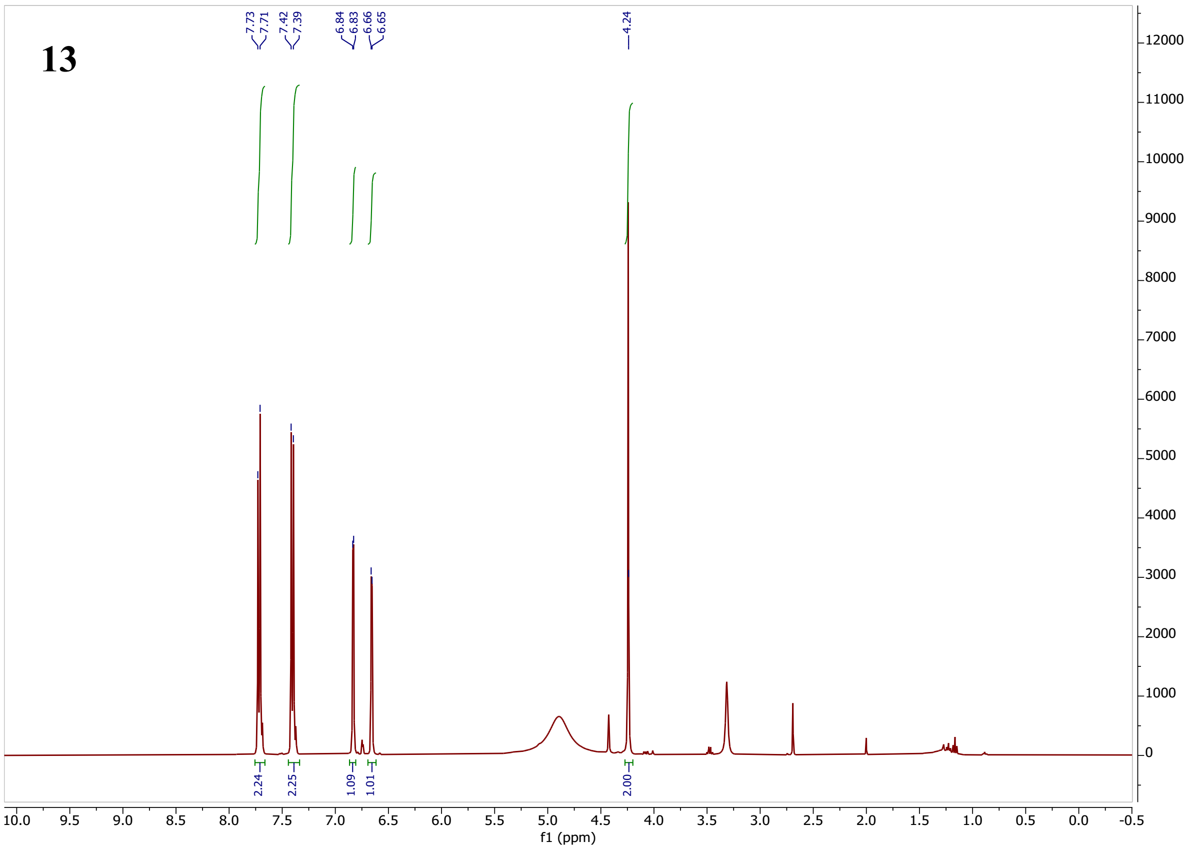

Figure S42. ^{1}H-NMR of isolated **13**.

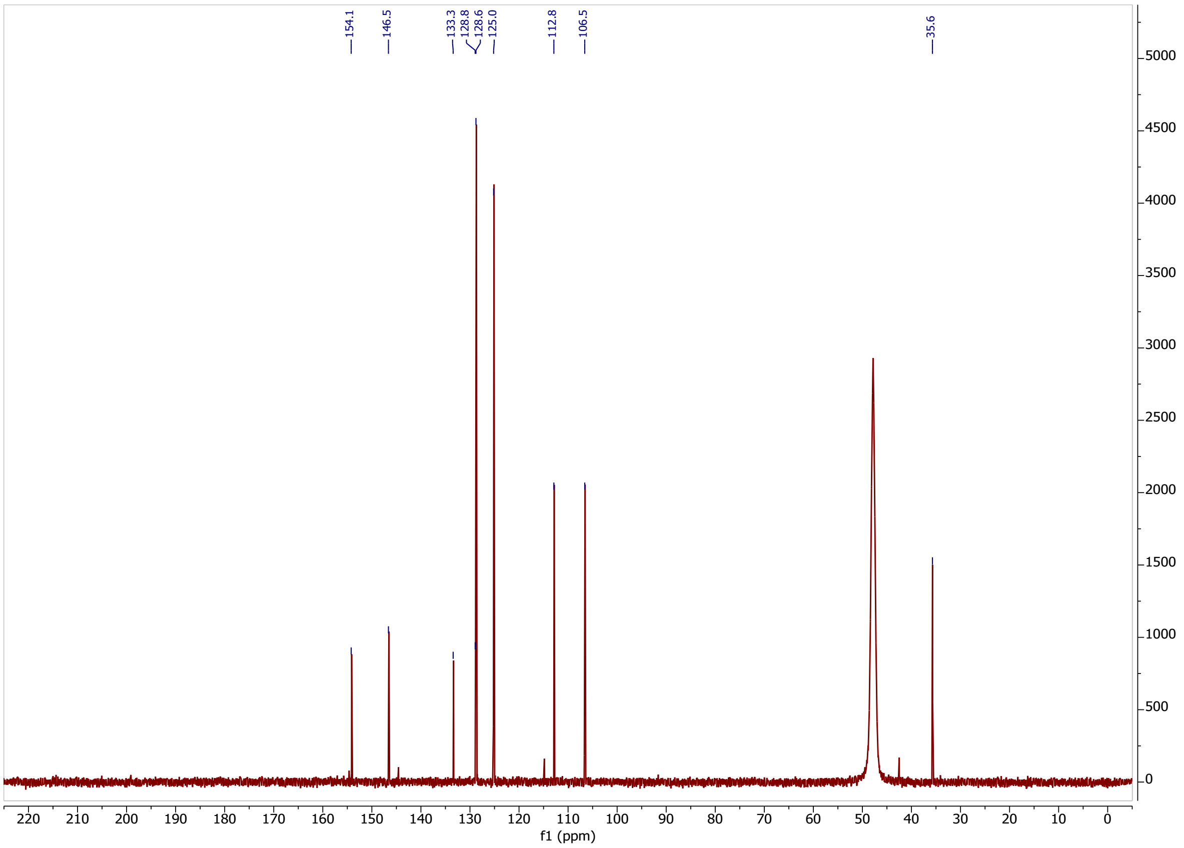

Figure S43. $^{13}C\{^{1}H\}$-NMR of isolated **13.**

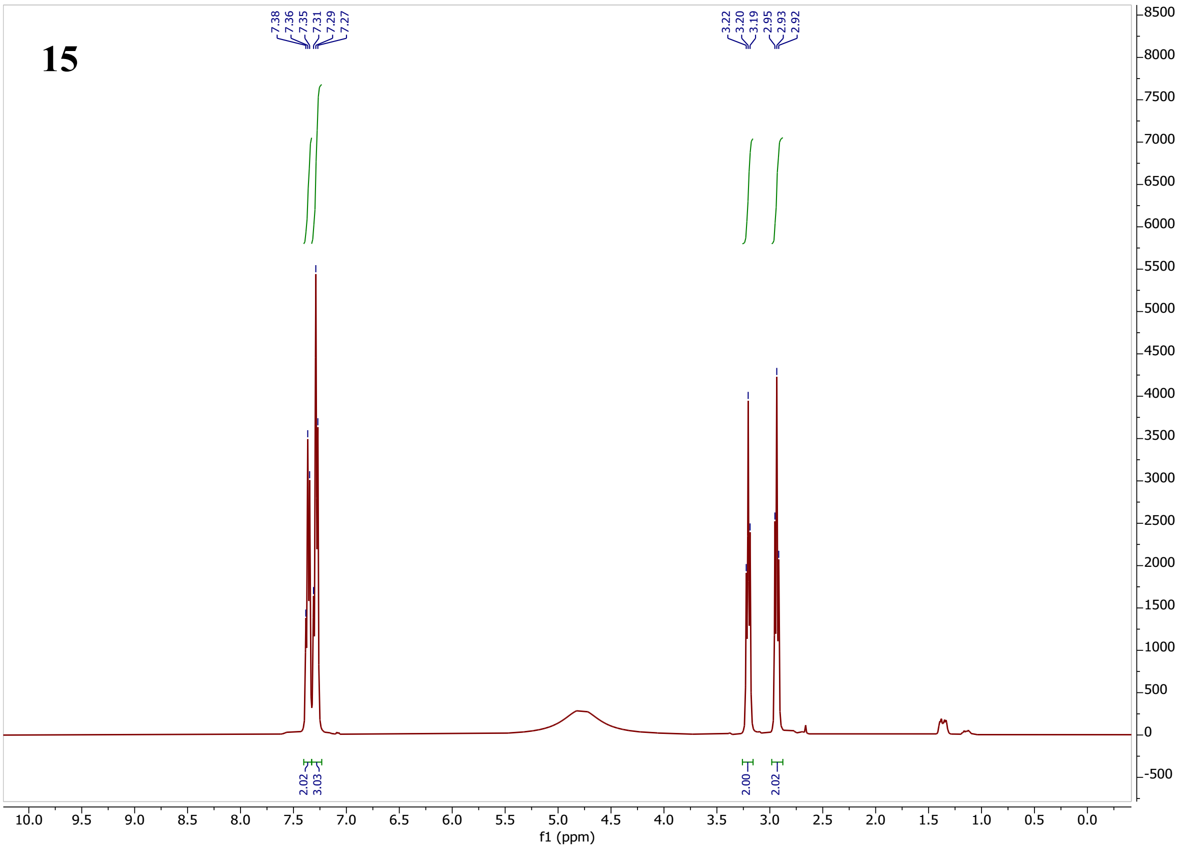

Figure S44. ^{1}H-NMR of isolated **15**.

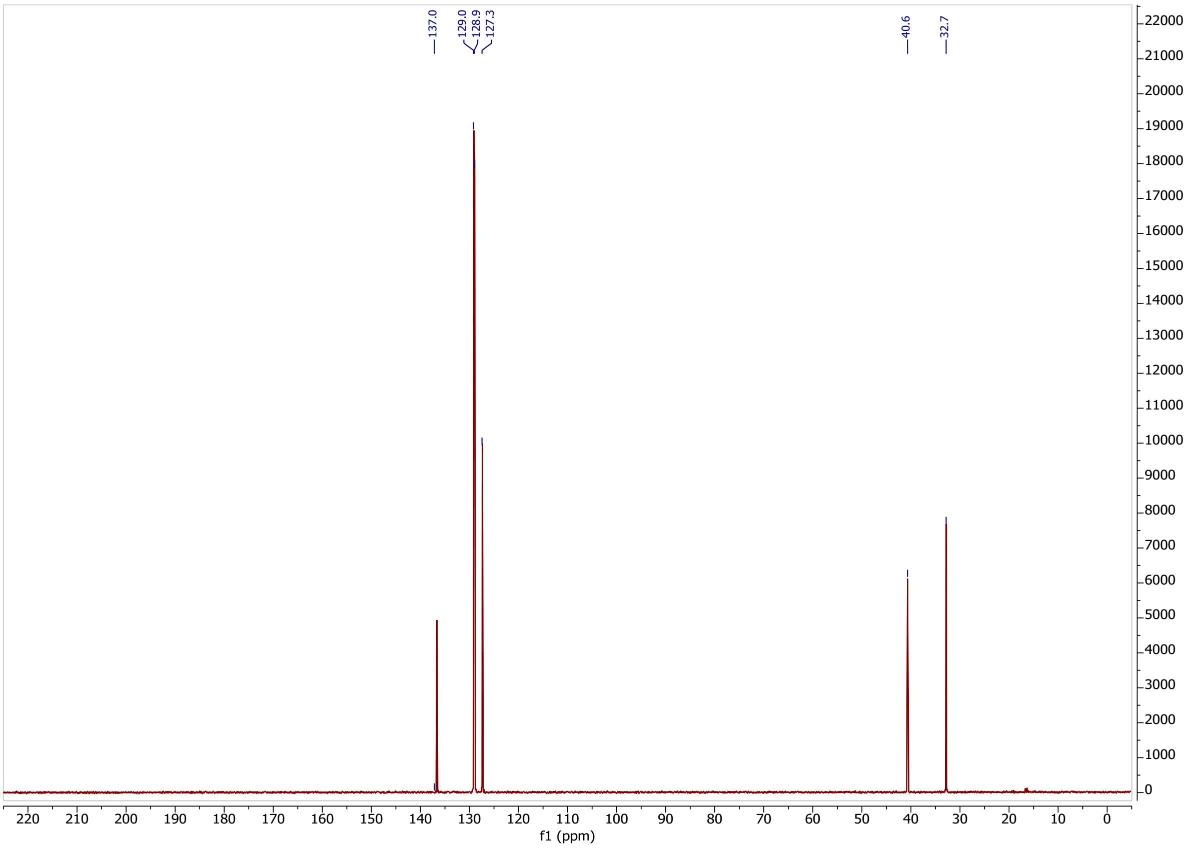

Figure S45. ^{13}C{^{1}H}-NMR of isolated **15.**

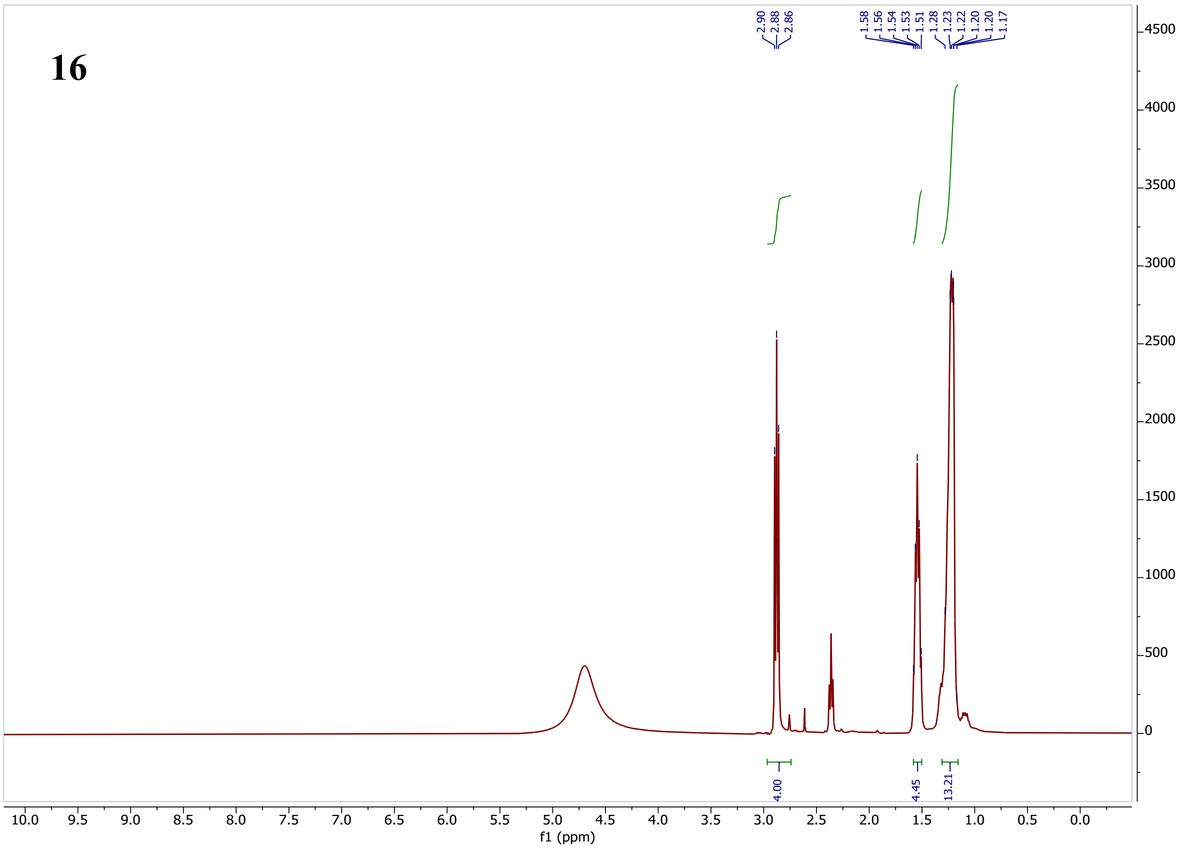

Figure S46. ^{1}H-NMR of isolated **16**.

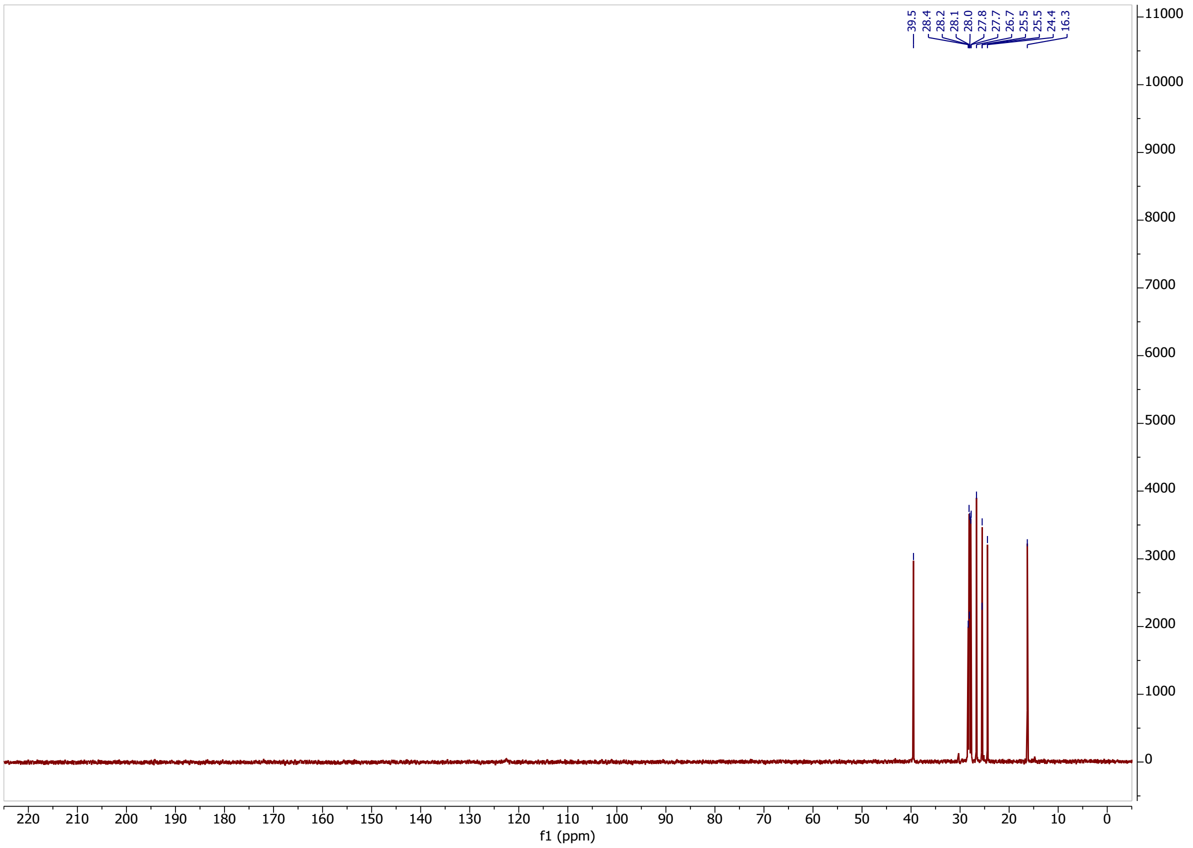

Figure S47. ^{13}C{^{1}H}-NMR of isolated **16.**

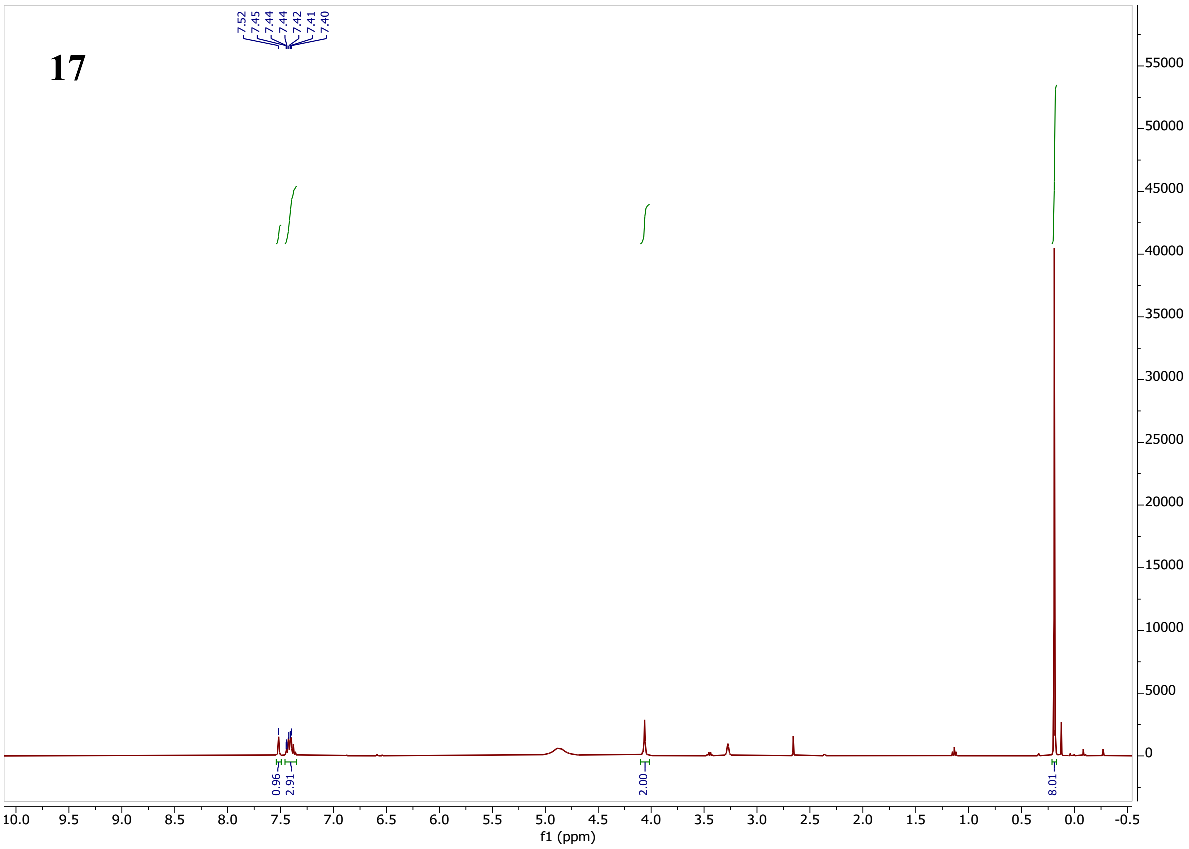

Figure S48. ¹H-NMR of isolated **17**.

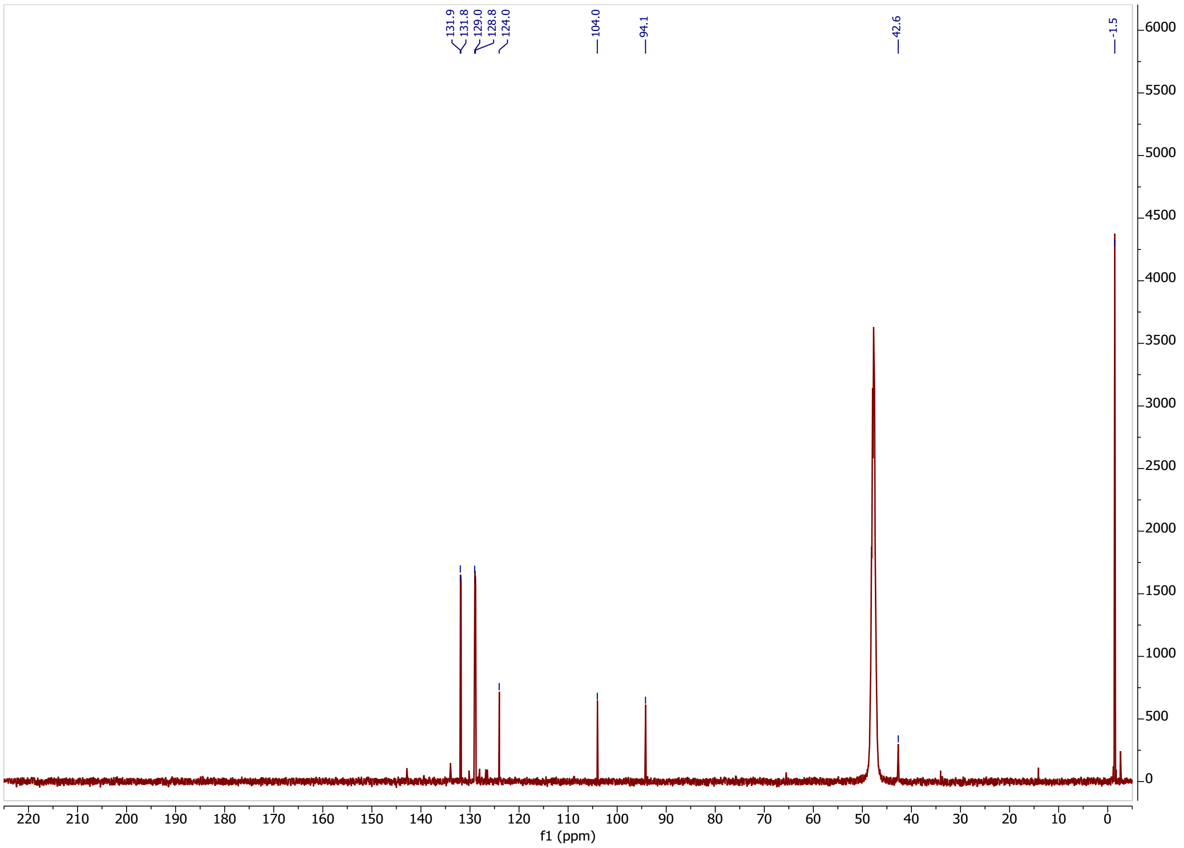

Figure S49. $^{13}C\{^{1}H\}$-NMR of isolated **17.**

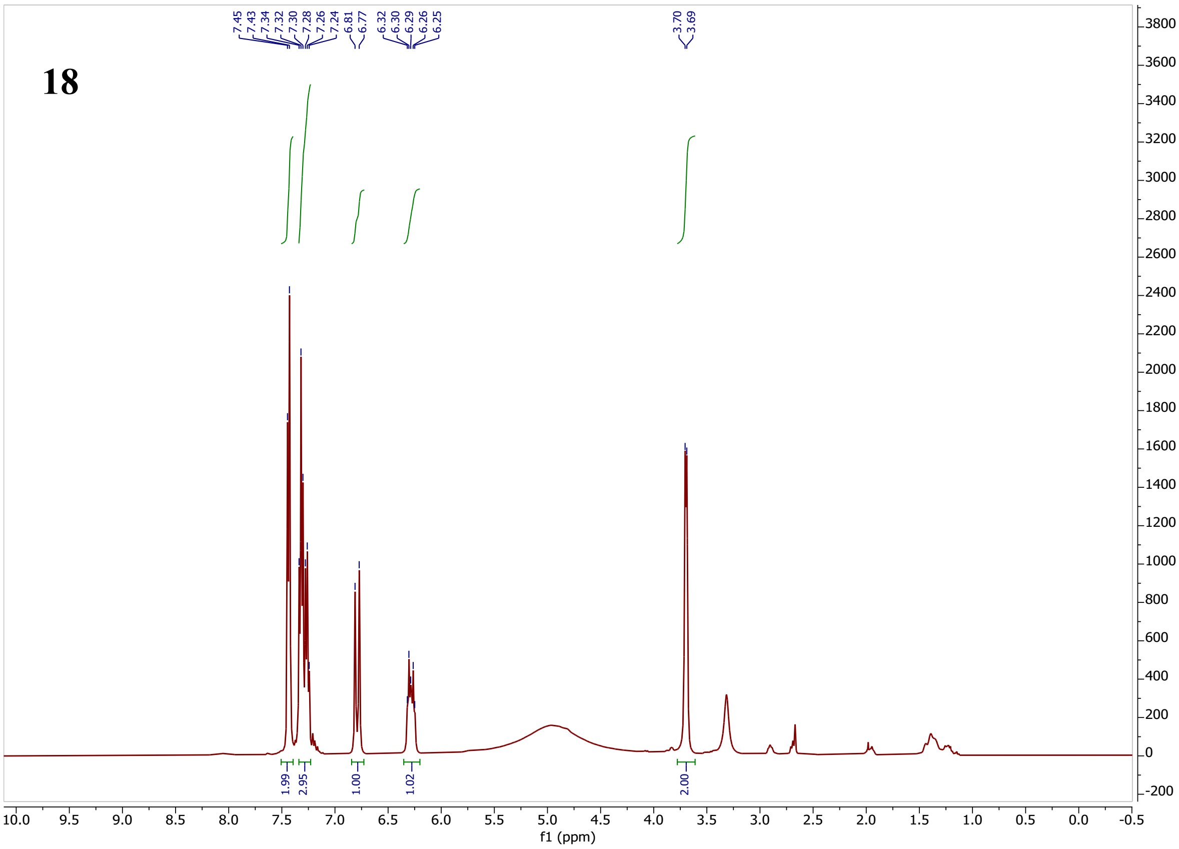

Figure S50. ^{1}H-NMR of isolated **18**.

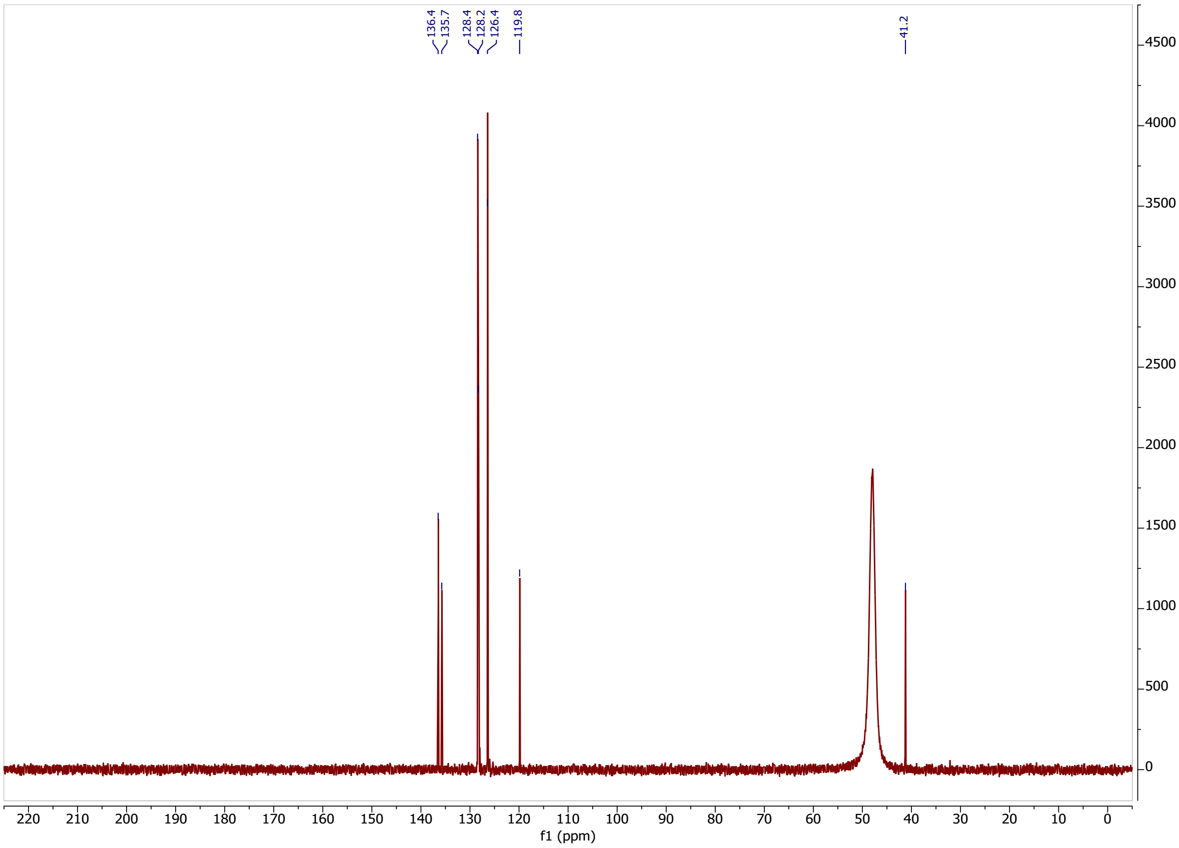

Figure S51. $^{13}C\{^{1}H\}$-NMR of isolated **18.**

7 References

(1) S. Weber, B. Stöger, L.F. Veiros and K. Kirchner, *ACS Catal.* 2019, **9**, 9715-9720.

(2) J.A. Garduño and J.J. Garcia, *ACS Catal.* 2018, **8**, 392-401.

(3) I. Blaha, S.Weber, R. Dülger, L.F. Veiros and K. Kirchner, ChemRxiv 2024, DOI: 10.26434/chemrxiv-2024-vvxlx.

(4) S. Weber, B. Stöger and K. Kirchner *Org.Lett.* 2018**, 20**, 7212-7215.

(5) S. Weber, L.F. Veiros and K. Kirchner, *Adv. Syn. Catal.* 2019, **361**, 5412-5420.

(6) N. Gautam, R. Logdi, S. P., N.M. Rajendran, A.K. Tiwariand S.K. Mandal, *Chem. Commun.*, 2022, **58**, 3047-3050

(7) B. Yan, X. Ma, Z. Pang and Z. Yang, *New J. Chem.*, 2023, **47**, 3202-3206.

(8) L.-M. Altman, M.C.D: Fürst, E. I. Gans, V. Zantop, G. Pratsch and M.R. Heinrich, *Org. Lett.* 2020, **22**, 479-482.
